# Integrated Microbiome and Metabolome Analysis Reveals Hypothalamic‐Comorbidities Related Signatures in Craniopharyngioma

**DOI:** 10.1002/advs.202400684

**Published:** 2024-09-03

**Authors:** Ben Lin, Zhen Ye, Zhan Cao, Zhao Ye, Yifei Yu, Weiliang Jiang, Sichen Guo, Vladimir Melnikov, Peng Zhou, Chenxing Ji, Chengzhang Shi, Zerui Wu, Zhengyuan Chen, Yihua Xu, Qilin Zhang, Zengyi Ma, Nidan Qiao, Long Chen, Xuefei Shou, Xiaoyun Cao, Xiang Zhou, Li Zhang, Min He, Yongfei Wang, Hongying Ye, Yiming Li, Zhaoyun Zhang, Meng Wang, Renyuan Gao, Yichao Zhang

**Affiliations:** ^1^ Department of Neurosurgery Huashan Hospital Fudan University Shanghai 200040 China; ^2^ Department of General Surgery Shanghai Tenth People's Hospital School of Medicine Tongji University Shanghai 200072 China; ^3^ Institute of Gut Microbiota Research and Engineering Development Shanghai Tenth People's Hospital Tongji University School of Medicine Shanghai 200072 China; ^4^ Department of Endocrinology and Metabolism Huashan Hospital Fudan University Shanghai 200040 China; ^5^ Department of Gastroenterology Shanghai General Hospital Shanghai Jiao Tong University School of Medicine Shanghai 200080 China; ^6^ Shanghai Key Laboratory of Pancreatic Disease Institute of Pancreatic Disease, Shanghai Jiao Tong University School of Medicine Shanghai 201620 China; ^7^ Shanghai Medical College Fudan University Shanghai 200032 China; ^8^ Department of Cardiology Huashan Hospital Fudan University Shanghai 200040 China; ^9^ National Center for Neurological Disorders Shanghai 200040 China; ^10^ Shanghai Key Laboratory of Brain Function and Restoration and Neural Regeneration Shanghai 200040 China; ^11^ Neurosurgical Institute of Fudan University Fudan University Shanghai 200040 China; ^12^ Shanghai Clinical Medical Center of Neurosurgery Shanghai 200040 China

**Keywords:** craniopharyngiomas, gut microbiota, hypothalamus‐pituitary axis, serum metabolome

## Abstract

Craniopharyngioma (CP) is an intracranial tumor with high mortality and morbidity. Though biologically benign, CP will damage the hypothalamus, inducing comorbidities such as obesity, metabolic syndrome, and cognitive impairments. The roles of gut microbiome and serum metabolome in CP‐associated hypothalamic comorbidities are aimed to be explored. Patients with CP are characterized by increased Shannon diversity, *Eubacterium*, *Clostridium*, and *Roseburia*, alongside decreased *Alistipes* and *Bacteroides*. CP‐enriched taxa are positively correlated with dyslipidemia and cognitive decline, while CP‐depleted taxa are negatively associated with fatty liver. Subsequent serum metabolomics identified notably up‐regulated purine metabolism, and integrative analysis indicated an association between altered microbiota and elevated hypoxanthine. Phenotypic study and multi‐omics analysis in the Rax‐CreER^T2^::Braf^V600E/+^::Pten^Flox/+^ mouse model validated potential involvement of increased *Clostridium* and dysregulated purine metabolism in hypothalamic comorbidities. To further consolidate this, intervention experiments are performed and it is found that hypoxanthine co‐variated with the severity of hypothalamic comorbidities and abundance of *Clostridium*, and induced dysregulated purine metabolism along with redox imbalance in target organs (liver and brain cortex). Overall, the study demonstrated the potential of increased *Clostridium* and up‐regulated purine metabolism as signatures of CP‐associated hypothalamic‐comorbidities, and unveiled that elevated *Clostridium*, dysregulated purine metabolism, and redox imbalance may mediate the development and progression of CP‐associated hypothalamic‐comorbidities.

## Graphical abstract

Craniopharyngioma (CP) is an intracranial tumor with prevalent hypothalamic comorbidities. The roles of gut microbiome and serum metabolome in CP‐associated hypothalamic comorbidities are aimed to be explored. The potential of increased *Clostridium* is demonstrated and purine metabolism is up‐regulated as signatures of CP‐associated hypothalamic‐comorbidities. And it is unveiled that elevated *Clostridium*, dysregulated purine metabolism, and redox imbalance may mediate the development and progression of CP‐associated hypothalamic comorbidities.

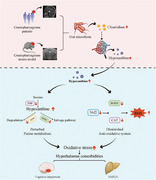

## 1 Introduction

Craniopharyngiomas (CPs) are tumors located in the pituitary sellar and suprasellar regions, arising from embryonic remnants of Rathke's pouch. CPs are among the most challenging intracranial lesions, with an annual incidence ranging from 0.05 to 0.25 per 100 000 individuals.^[^ ^1^ ^]^ The unknown molecular pathogenesis of CP involves somatic mutations in CTNNB1 and BRAF‐V600E.^[^ ^2^ ^]^ Clinical manifestations of CP are aggressive and characterized by high recurrence rates and long‐term mortality.^[^ ^3^ ^]^ Until now, the primary treatment for CP remains to be neurosurgery.^[^ ^4^ ^]^ However, it is worth noting that despite recent progress in surgical techniques and endocrinological care, which have improved perioperative mortality and complete tumor resection rates, most of the patients with CP are suffering from hypothalamic comorbidities.^[^ ^5^ ^]^ Due to the hypothalamic involvement and treatment‐associated hypothalamic lesions, patients with CP are at high risk develop hypopituitarism, hyperphagia, disturbance of circadian rhythms, and metabolic syndrome, subsequently resulting in deficits in physiological, emotional, and social function, finally leading to reduction of quality of life.^[^ ^6^ ^]^

Gut microbiota has been proved critical for an array of diseases, including gastrointestinal ailments,^[^ ^7^ ^]^ metabolic disturbances (obesity^[^ ^8^ ^]^ and diabetes^[^ ^9^ ^]^), cancer,^[^ ^10^ ^]^ and neurodegenerative disorders.^[^ ^11^ ^]^ Gut microbiota‐produced metabolites can access the bloodstream via absorption and enterohepatic circulation or directly permeate an impaired gut barrier.^[^ ^12^ ^]^ Remarkably, up to one‐third of the small molecules circulating in human blood originate from gut bacteria.^[^ ^13^ ^]^ Our previous review concluded three interaction paradigms of gut‐brain axis in brain tumors.^[^ ^14^ ^]^ To be more specific, gut microbiota and related metabolites regulate the hypothalamic appetite,^[^ ^15^ ^]^ reproductive development,^[^ ^16^ ^]^ menstrual cycle,^[^ ^17^ ^]^ and clock gene expression.^[^ ^18^ ^]^ However, the characteristics of gut microbiota and the association between hypothalamic‐comorbidities and the alteration in microbial metabolic products in craniopharyngioma remain elusive.

**Figure 1:**
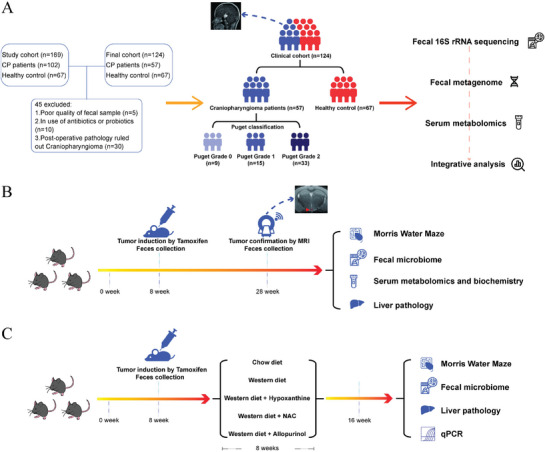
Workflow of the study. A) Initially, a cohort of 102 patients with craniopharyngioma (CP) and 67 healthy controls (HC) were recruited. After applying filtration criteria, the final cohort included 57 patients with CP and 67 HC. Patients with CP were further categorized based on the degree of hypothalamic involvement. Participants underwent fecal 16S rRNA gene sequencing, fecal metagenomics, and serum metabolomics analyses, followed by the bioinformatic integration of gut microbiome and serum metabolome data. B) To complement the clinical data, a transgenic animal model of CP was employed. Tumor formation was induced in the 8th week, and tumor presence was confirmed in the 28th week in the rodent models. Fecal samples were collected at both time points. Subsequently, behavioral assessments using the Morris water maze, along with fecal microbiome analysis, serum metabolomics, serum biochemistry, and liver histopathological analysis, were sequentially conducted in the rodent models. C) To inspect the potential influence on CP‐related hypothalamic comorbidities from perturbed purine metabolism, we treated CP mouse model with a chow diet, western diet, western diet + hypoxanthine (Hx), western diet + N‐Acetyl Cysteine (NAC) and western diet + allopurinol (Allo) respectively for 8 weeks. Thereafter, Morris water maze, fecal microbiome analysis, liver histopathological analysis, and qPCR of target organs (liver and brain cortex), were sequentially conducted.

Hence, we conducted the study to elucidate the potential pathophysiological implications of the gut microbiome and serum metabolites in CP‐associated hypothalamic comorbidities. Our study spanned both clinical patients and transgenic mouse models of CP. In the clinical part (**Figure** **1**A), we took advantage of 16S rRNA sequencing, metagenomics, and high‐performance liquid chromatography‐mass spectrometry (HPLC‐MS) to inspect the shifts in the gut microbiome and serum metabolome of patients with CP. In our animal experiments (Figure 1B), we first consolidated the CP‐specific perturbations in gut microbiome and serum metabolome, along with their association with CP‐associated hypothalamic comorbidities. At last, we carried out interventions experiments to manipulate the concentration of hypoxanthine, thereby delineating the relations between perturbed purine metabolism, gut microbiome, and CP‐associated hypothalamic comorbidities (Figure 1C).

## 2 Results

### 2.1 Clinical Characteristics of Enrolled Subjects

After filtration, the final cohort of this study comprised 57 patients, along with 67 (Table S1, Supporting Information) and 52 healthy controls, HC (Table S2, Supporting Information) for 16S rRNA and metagenomic analysis respectively (Figure 1A). Baseline characteristics of the 57 patients with CP included in the study were provided in materials (Table S3, Supporting Information). Notably, the detection rate of fatty liver reached 48.48%, surpassing the prevalence observed in the general population.^[^ ^19^ ^]^

To explore the pathological consequences of hypothalamic‐pituitary injury associated with CP, patients with CP were stratified according to degree of hypothalamic involvement: Puget Grade 0 (n = 9), Puget Grade 1 (n = 15), and Puget Grade 2 (n = 33). Comparison of clinical characteristics among these subgroups (**Table** **1**) revealed notable increases in aspartate transaminase (*p* = 0.042), 2 hour postprandial insulin (*p* = 0.002), and 2 hour postprandial C peptide (*p* = 0.002) in the Puget Grade 2 group, suggesting a positive association between the extent of hypothalamic involvement, liver injury, and abnormal glucose metabolism. Neurocognitive questionnaires (Short Form 36, SF‐36 and Symptom Check List‐90, SCL90) were obtained from 27 patients, with detailed parameters presented in Table S4 (Supporting Information). Compared with established Chinese norm data of SF‐36^[^ ^20^ ^]^ and SCL90,^[^ ^21^ ^]^ significantly lower scores in physical functioning, role physical, bodily pain, vitality, role emotional, and mental health were observed in CP patients. Meanwhile, there is no remarkable difference in neurocognitive questionnaires between subgroups (Puget grades 1 and 2) of CP patients. These results indicate that the neurocognitive performance was notably impaired in CP patients.

**Table 1:** Clinical indices of 57 patients with craniopharyngioma among different Puget grades.

	Puget 0 (n = 9)	Puget 1 (n = 15)	Puget 2 (n = 33)	*p* value
Demographic features				
Female (n, %)	3, 33.33%	8, 53.33%	10, 30.30%	0.300
Age (years)	42.7 ± 6.069	44.5 ± 4.745	43.8 ± 2.726	0.966
BMI (kg/cm2)	24.3 ± 1.334	24.1 ± 1.165	25.0 ± 0.728	0.771
SBP (mmHg)	119.4 ± 4.738	118.6 ± 5.191	119.4 ± 2.331	0.737
DBP (mmHg)	81.1 ± 3.494	73.7 ± 3.010	76.5 ± 0.730	0.193
Tumor Features				
ACP (n, %)	5, 55.55%	13, 86.67%	25, 75.76%	0.018^‡^
PCP (n, %)	2, 22.22%	2, 13.33%	8, 24.24%	
Cystic Lesions (n, %)	2, 22.22%	0, 0.00%	0, 0.00%	
Ki67 (%)	3.3 ± 0.898	3.0 ± 0.338	3.8 ± 0.358	0.439
Fatty liver (Puget 0 = 7, Puget 1 = 9, Puget 2 = 17)	3, 42.86%	6, 66.67%	7, 41.18%	
Laboratory testing				
WBC(x10^9/L)	5.5 ± 0.452	5.8 ± 0.281	6.7 ± 0.282	0.041^‡^
NEU (%)	2.7 ± 0.378	3.2 ± 0.226	3.9 ± 0.201	0.015^‡^
LYM (%)	2.2 ± 0.281	2.0 ± 0.165	2.2 ± 0.116	0.615
MON (%)	0.3 ± 0.019	0.3 ± 0.026	0.4 ± 0.024	0.013^‡^
ALP (U/L)	78.2 ± 15.635	65.9 ± 5.738	69.5 ± 3.276	0.522
ALB (g/L)	43.7 ± 1.312	44.3 ± 0.859	44.3 ± 0.639	0.893
TBIL (µmol/L)	10.8 ± 2.485	10.4 ± 1.764	7.9 ± 0.471	0.250
UA (mmol/L)	0.3 ± 0.028	0.3 ± 0.029	0.3 ± 0.018	0.629
DBIL (µmol/L)	5.2 ± 2.257	3.6 ± 0.521	3.3 ± 0.187	0.512
BUN (mmol/L)	5.0 ± 0.374	4.3 ± 0.272	4.5 ± 0.292	0.587
CRE (µmol/L)	71.9 ± 5.425	71.9 ± 4.913	70.6 ± 2.502	0.957
GGT (U/L)	25.6 ± 8.219	24.5 ± 3.900	53.3 ± 10.567	0.059
TPRO (g/L)	67.1 ± 1.419	68.2 ± 1.696	69.0 ± 0.841	0.606
ALT (U/L)	21.8 ± 3.673	25.2 ± 3.921	44.3 ± 9.384	0.207
AST (U/L)	19.1 ± 1.975	21.8 ± 1.873	31.8 ± 4.320	0.042^‡^
HbA1c (%)	5.7 ± 0.138	5.7 ± 0.128	5.8 ± 0.129	0.890
FPG (mmol/L)	4.8 ± 0.125	4.8 ± 0.155	5.0 ± 0.123	0.485
Fasting Insulin (mU/L)	7.6 ± 1.141	9.5 ± 2.128	19.0 ± 5.435	0.236
Fasting C peptide (ug/L)	2.0 ± 0.197	2.1 ± 0.289	3.0 ± 0.346	0.102
2hPG (mmol/L)	6.7 ± 0.846	8.7 ± 1.491	8.8 ± 0.604	0.303
2 h INS (mU/L)	41.7 ± 4.377	62.2 ± 14.141	143.8 ± 24.944	0.002^‡*^
2 h C peptide (ug/L)	7.6 ± 0.546	7.6 ± 1.084	12.4 ± 1.120	0.002^‡*^
TG (mmol/L)	1.5 ± 0.343	1.4 ± 0.179	2.3 ± 0.354	0.199
CHO (mmol/L)	4.6 ± 0.364	4.9 ± 0.495	5.4 ± 0.229	0.268
sdLDL (mmol/L)	2.8 ± 0.384	3.4 ± 0.439	3.5 ± 0.200	0.386
HCY (µmol/L)	12.5 ± 2.077	10.1 ± 0.816	13.2 ± 1.502	0.386
GH (ng/mL)	0.4 ± 0.133	0.4 ± 0.121	0.4 ± 0.823	0.983
IGF‐1 index	0.5 ± 0.052	0.4 ± 0.040	0.5 ± 0.041	0.412
ACTH (pg/mL)	49.3 ± 9.573	53.3 ± 9.567	55.2 ± 6.382	0.909
TSH (mIU/L)	2.1 ± 0.323	2.1 ± 0.443	1.7 ± 0.179	0.401
TT4 (nmol/L)	96.1 ± 7.971	87.5 ± 5.663	88.4 ± 2.612	0.498
TT3 (nmol/L)	1.5 ± 0.146	1.4 ± 0.088	1.5 ± 0.057	0.847
FT4 (pmol/L)	14.9 ± 1.431	12.4 ± 0.674	12.8 ± 0.492	0.131
FT3 (pmol/L)	4.3 ± 0.439	3.9 ± 0.210	4.2 ± 0.141	0.405
FSH (IU/L)	7.7 ± 4.126	7.4 ± 2.215	5.0 ± 0.906	0.393
LH (IU/L)	5.0 ± 1.969	3.8 ± 1.056	3.6 ± 0.598	0.660
PRL (ng/mL)	29.8 ± 8.968	39.1 ± 7.941	40.3 ± 6.663	0.722
DHEA (µmol/L)	4.1 ± 1.153	4.1 ± 0.634	6.5 ± 0.763	0.079
COR (ug/dl)	10.2 ± 1.782	11.8 ± 1.994	11.2 ± 1.137	0.840
E2 (pmol/L)	74.4 ± 13.481	68.8 ± 9.710	87.0 ± 7.600	0.343
T (nmol/L)	7.3 ± 2.724	4.0 ± 1.508	5.3 ± 1.196	0.526
PRO (nmol/L)	0.6 ± 0.047	0.8 ± 0.154	0.6 ± 0.054	0.150

SBP: systolic blood pressure; DBP: diastolic blood pressure; WBC: white blood cell; NEU: neutrophil; LYM: lymphocyte; MON: monocyte; ALP: alkaline phosphatase; ALB: albumin; TBIL: total bile acid; UA: uric acid; DBIL: direct bile acid; BUN: blood urea nitrogen; CRE: creatinine; GGT: Gamma‐glutamyl transpeptidase; TPRO: total protein; ALT: alanine transaminase; AST: aspartate transaminase; HbA1c: Hemoglobin A1c; FPG: fasting plasma glucose; 2hPG: 2‐hour postprandial plasma glucose; 2hINS: 2‐hour postprandial insulin; 2 h C peptide: 2‐hour postprandial C‐peptide; TC: triglyceride; CHO: cholesterol; sdLDL: small dense low density lipoprotein; HCY: homocysteine; GH: growth hormone; IGF‐1: insulin like growth factor 1; ACTH: adrenocorticotropic hormone; TSH: thyroid stimulating hormone; T4: total thyroxine; T3: total triiodothyronine; FT4: free thyroxine; FT3: free triiodothyronine; LH: luteinizing hormone; PRL: prolactin; DHEA: dehydroepiandrosterone; COR: cortisol; FSH: follicle stimulating hormone; E2: estradiol; T: testosterone; P: progesterone; PRO: progesterone. ‡*p* < 0.05 between p0 versus p1; ‡*p* < 0.05 between p0 versus p2; *p < 0.05 between p1 versus p2.

### 2.2 Differences in Gut Microbiome Between Patients With CP and HC

In 16S rRNA analysis, the patients (CP group, n = 57) and HC (HC group, n = 67) were well‐matched in terms of age (*p* = 0.051) and body mass index, BMI (*p* = 0.263). Regarding microbial diversity between patients and HC, we observed that 713 of the 1070 OTUs were shared between patients with CP (CP group) and HC (HC group). Conversely, 253 and 104 operational taxonomic units (OTUs) were exclusive to the CP and HC group respectively (Figure S1A, Supporting Information). Overall, there were no significant inter‐group differences in alpha diversity in the fecal microbiota, as indicated by similar Chao 1 index (*p* = 0.64), good coverage diversity (*p* = 0.39), phylogenetic diversity whole tree diversity (*p* = 0.82), observed species diversity (*p* = 0.69), Shannon index (*p* = 0.077), and Simpson index (*p* = 0.053) (Figure S1B, Supporting Information). However, significant differences in beta diversity between the CP and HC groups were observed (Figure S2A,B, Supporting Information). The analysis of similarities (ANOSIM test) confirmed a significant difference between the two groups (R = 0.086, *p* = 0.001) (Figure S2A, Supporting Information), and PERMANOVA analysis further substantiated altered microbial communities in patients with CP (R^2^ = 0.042, ADONIS function *p* = 0.001) (Figure S2B, Supporting Information). To assess the influence of baseline factors on microbial composition, an redundancy analysis (RDA) was conducted, revealing a non‐significant effect (*p* = 0.091) from the combination of these baseline indices (Figure S2C, Supporting Information). Nonetheless, individual indices indicated that age (R^2^ = 0.068, *p* = 0.024) and BMI (R^2^ = 0.099, *p* = 0.001) significantly contributed to the clustering effect.

After adjusting for age and BMI using Multivariate Association with Linear Models (MaAslin2), we explored the microbial composition of these two groups. *Firmicutes* and *Bacteroidetes* were abundant in both groups (Figure S1C, Supporting Information). The microbial compositions at the genus level in the two groups are shown in Figure S1D (Supporting Information). Further differentiation analysis revealed that the genera *Faecalibacterium* (*p* < 0.001) and *Clostridium XIVa* (*p* = 0.004) were significantly enriched in the CP group compared to the HC group. In contrast, the abundances of the genera *Blautia* (*p* < 0.001) and *Romboutsia* (*p* < 0.001) were significantly higher in the HC group than in the CP group (Figure S2D, Supporting Information).

### 2.3 Taxonomic and Functional Characterization of CP Microbiome

To gain a comprehensive understanding of taxonomic alterations and functional deviations in patients with CP, we conducted shotgun metagenomic sequencing on 56 patients with CP and 52 HC. Age was well‐matched between the two groups (*p* = 0.291), while BMI in the CP group was slightly higher than in the HC group (CP vs HC: 24.69 ± 4.21 kg m^−2^ vs 23.08 ± 3.19 kg m^−2^, *p* = 0.028).

**Figure 2:**
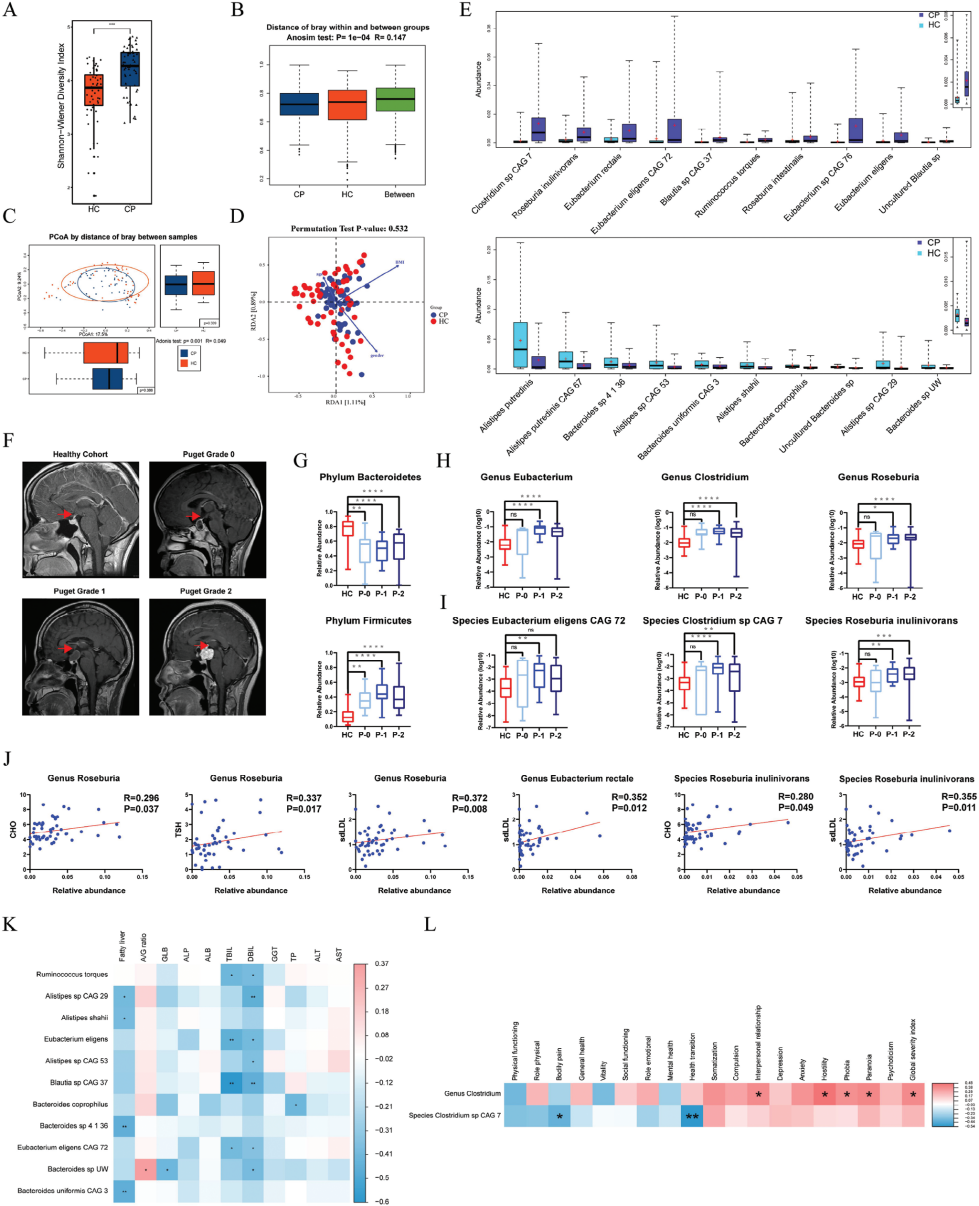
Altered gut microbiome was associated with hypothalamic comorbidities in patients with CP. A) Alpha diversity (Shannon–Wiener index) at species level was different between patients with craniopharyngioma (CP, n = 56) and healthy controls (HC, n = 52) (*p* < 0.001). B) ANOSIM and C) ADONIS revealed significantly altered Beta diversity at the species level. D) RDA analysis and permutation test demonstrated non‐significant confounding effects of baseline information (*p* = 0.532). E) Wilcoxon rank‐sum test identified altered species in patients with CP (purple) and HC (blue), displaying the top 10 most abundant species in each group. F) MRI images of HC and patients with CP with different Puget classifications (P‐0, P‐1, and P‐2), highlighting the hypothalamus area with arrows. G) Relative abundance of phylum Bacteroidetes and Firmicutes among HC and patients with CP with different Puget classifications. H) Abundance of Eubacterium, Clostridium, and Roseburia in different subgroups. I) Abundance of Eubacterium eligens CAG 72, Clostridium sp. CAG 7, and Roseburia inulinivorans in different subgroups. J) Significant correlations were observed between Roseburia and CHO (*r* = 0.296, *p* = 0.037), TSH (*r* = 0.337, *p* = 0.017), sdLDL (*r* = 0.372, *p* = 0.008), and between Eubacterium rectale and sdLDL (*r* = 0.352, *p* = 0.012). Noteworthy correlations were found between Roseburia inulinivorans and CHO (*r* = 0.280, *p* = 0.049) and sdLDL (*r* = 0.355, *p* = 0.011). K) CP‐depleted taxa (Bacteroides uniformis CAG 3: *r* = −0.460, *p* = 0.008; Bacteroides sp. 4 1 36: *r* = −0.460, *p* = 0.008; Alistipes shahii: *r* = −0.393, *p* = 0.026; Alistipes sp. CAG 29: *r* = −0.399, *p* = 0.024) showed negative associations with the occurrence of fatty liver. L) Clostridium exhibited positive correlations with scores of interpersonal relationship (*r* = 0.394, *p* = 0.041), hostility (*r* = 0.479, *p* = 0.012), phobia (*r* = 0.384, *p* = 0.048), paranoia (*r* = 0.415, *p* = 0.031), and global severity index (*r* = 0.404, *p* = 0.037), while Clostridium sp. CAG 7 was negatively correlated with scores of bodily pain (*r* = −0.404, *p* = 0.037) and health transition (*r* = −0.540, *p* = 0.004). *p < 0.05, **p < 0.01, ***p < 0.001, ****p < 0.0001.

At the genus level, alpha diversity, as measured by the Shannon–Wiener diversity index, was significantly higher in the CP group (Figure S3A, Supporting Information), and beta diversity, assessed using the ANOSIM test (R = 0.149, *p* < 0.001), and the ADONIS test (R = 0.084, *p* = 0.001), exhibited significant differences between the two groups (Figure S3B,C, Supporting Information). Consistently, alpha diversity at the species level (Shannon–Wiener diversity index) was also significantly higher in the CP group (**Figure** **2**A), with significant differences in beta diversity at the species level demonstrated by the ANOSIM (R = 0.147, *p* < 0.001, Figure 2B) and ADONIS tests (R = 0.049, *p* = 0.001, Figure 2C).

We further examined the microbial composition at the phyla and genera levels (Figure S3E, Supporting Information). A comparison of microbial profiles at the genus level revealed 1564 genera shared between the two groups, with 96 genera and 332 genera exclusive to the CP and HC groups, respectively (Figure S3D, Supporting Information).

To evaluate the potential influence of baseline factors on microbial composition, we conducted an RDA analysis. The permutation test indicated a non‐significant effect (*p* = 0.532) from the combination of these baseline indices (Figure 2D). However, inspection of individual indices revealed that sex (R^2^ = 0.091, *p* = 0.01) and BMI (R^2^ = 0.167, *p* = 0.001) significantly contributed to the clustering effect. After adjusting for sex and BMI using MaAslin (Tables S5 and S6, Supporting Information), we employed the Wilcoxon rank‐sum test to identify distinctive taxonomic differences between patients with CP and HC. At the genus level, *Eubacterium* (*p* < 0.001), *Clostridium* (*p* < 0.001), and *Roseburia* (*p* < 0.001) were more abundant in the CP group, while *Alistipes* (*p* < 0.001), *Prevotella* (*p* = 0.007), and *Tannerella* (*p* = 0.034) were predominant in HC (Figure S3F, Supporting Information). At the species level, analysis revealed that *Clostridium sp. CAG 7* (*p* < 0.001), *Roseburia inulinivorans* (*p* < 0.001), *Eubacterium rectale* (*p* = 0.005), and *Eubacterium eligens CAG 72* (*p* = 0.009) were enriched in the CP group. In contrast, *Alistipes putredinis* (*p* < 0.001), *A. putredinis CAG 67* (*p* < 0.001), *Bacteroides sp. 4_1_36* (*p* = 0.025), and *Alistipes sp. CAG 53* (*p* = 0.005) were more abundant in HC (Figure 2E).

To explore potential interactions between altered gut microbiota and hypothalamic involvement in the CP group, we compared microbial profiles among different Puget classifications. Typical sagittal contrast‐enhanced brain magnetic resonance imaging (MRI) images of patients with different Puget classifications are shown in Figure 2F. Compared to the HC group, the CP group exhibited a higher abundance of the phylum *Firmicutes* (*p* < 0.0001) and a lower abundance of the phylum *Bacteroidetes* (*p* < 0.0001), with no significant differences among the three subgroups (Figure 2G). Furthermore, in comparison to the HC group, the Puget Grade 1 and 2 groups displayed a higher abundance of the genera *Eubacterium* (*p* < 0.0001), *Clostridium* (*p* < 0.0001), and *Roseburia* (*p* = 0.0001), while there was no significant difference between the HC and Puget Grade 0 groups (Figure 2H). Consistent with the genus‐level findings, *E. eligens CAG 72* was more prevalent in the Puget Grade 1 group than in the HC group (*p* = 0.0097, Figure 2I), while there was no difference between the HC group and either the Puget Grade 0 or Grade 2 groups. Additionally, compared to the HC group, more *Clostridium sp. CAG 7* (*p* < 0.0001) and *R. inulinivorans* (*p* = 0.0001) were observed in fecal samples from patients with CP in the Grade 1 and 2 Puget groups (Figure 2I). These findings indicate that a higher abundance of the genera *Eubacterium*, *Clostridium*, and *Roseburia* in patients with CP is correlated with hypothalamic involvement.

In our exploration of functional differences and annotated pathways between patients with CP and HC (Figure S4A, Supporting Information), ATP‐binding cassette‐2 type transport system ATP‐binding protein (*p* < 0.001), RNA polymerase sporulation‐specific sigma factor (*p* < 0.001) and L‐serine dehydratase [EC:4.3.1.17] (*p* = 0.025) were enriched in patients with CP. Conversely, HC exhibited enrichment in iron complex transport system permease protein (*p* < 0.001), membrane fusion protein, multidrug efflux system (*p* = 0.008), and beta‐glucosidase [EC:3.2.1.21] (*p* = 0.043). Furthermore, after enrichment analysis (Figure S4B, Supporting Information), we observed that the CP group had a higher number of KO annotations related to carbohydrate metabolism (87 vs 42), global and overview maps (67 vs 28), amino acid metabolism (57 vs 30), energy metabolism (50 vs 29), metabolism of cofactors and vitamins (47 vs 13), nucleotide metabolism (26 vs 13), and lipid metabolism (12 vs 5). These findings suggest that the microbiota of patients with CP may exhibit a higher metabolic potential in these biological reactions.

To uncover potential pathophysiological implications of the altered gut microbiome in CP, we conducted Spearman's correlation analysis between metagenomic data and clinical indices. As shown in Figure 2J, we identified positive correlations between genus *Roseburia* and CHO (*r* = 0.296, *p* = 0.037), TSH (*r* = 0.337, *p* = 0.017), and sdLDL (*r* = 0.372, *p* = 0.008). Additionally, *E. rectale* showed a positive association with sdLDL (*r* = 0.352, *p* = 0.012). At the species level, *R. inulinivorans* was positively associated with CHO (*r* = 0.280, *p* = 0.049) and sdLDL (*r* = 0.355, *p* = 0.011). When examining the potential link between liver injury and the gut microbiome in CP (Figure 2K), our correlation analysis in individuals with abdominal ultrasound data revealed that CP‐depleted taxa (*Bacteroides uniformis CAG 3*: *r* = −0.460, *p* = 0.008; *Bacteroides sp. 4 1 36*: *r* = −0.460, *p* = 0.008; *Alistipes shahii*: *r* = −0.393, *p* = 0.026; *Alistipes sp. CAG 29*: *r* = −0.399, *p* = 0.024) were negatively associated with the presence of fatty liver. Regarding neurocognitive performance (Figure 2L), our association study in individuals with neurocognitive evaluation data demonstrated that genus *Clostridium* was positively correlated with scores of interpersonal relationship (*r* = 0.394, *p* = 0.041), hostility (*r* = 0.479, *p* = 0.012), phobia (*r* = 0.384, *p* = 0.048), paranoia (*r* = 0.415, *p* = 0.031), and the global severity index (*r* = 0.404, *p* = 0.037). Meanwhile, *Clostridium sp. CAG 7* was negatively correlated with scores of bodily pain (*r* = −0.404, *p* = 0.037) and health transition (*r* = −0.540, *p* = 0.004).

Taken together, these results suggest that patients with CP possess a distinct gut microbiome that may play a role in hypothalamic comorbidities, including impaired neurocognition and metabolic dysfunction, such as dyslipidemia and fatty liver.

### 2.4 Serum Metabolomic Differences Between Patients with CP and HC

To investigate CP‐specific metabolites with potential pathophysiological implications, we conducted HPLC‐MS to analyze serum metabolite profiles, enrolling patients with CP (n = 52) and matched HC (n = 50) in terms of age (*p* = 0.182) and BMI (*p* = 0.093).

**Figure 3:**
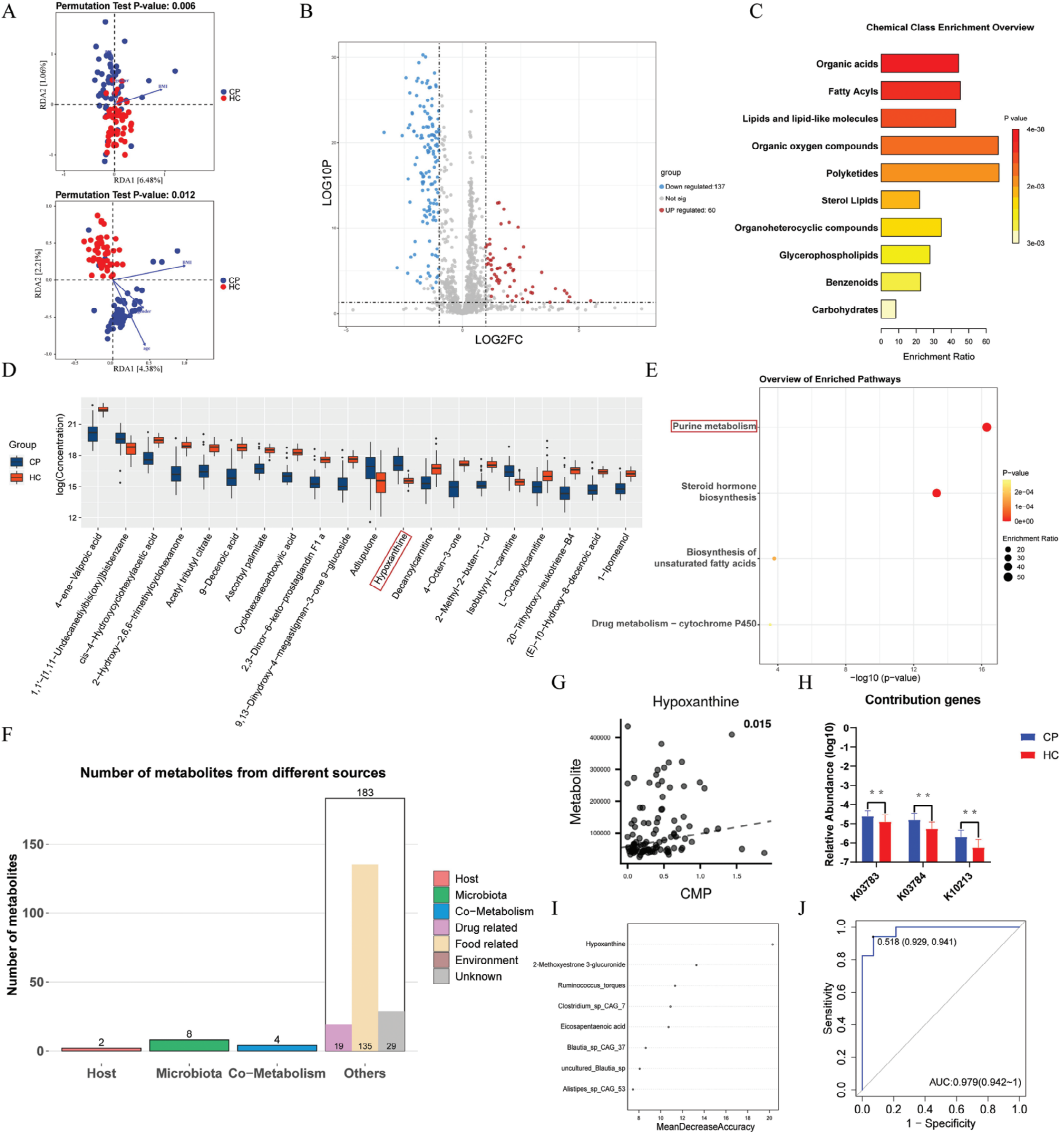
Aberrant metabolome in patients with CP and its interaction with gut microbiome. A) RDA was conducted to evaluate the potential confounding effect of basic information, and the permutation test indicated a significant effect (positive‐mode: *p* = 0.006; negative‐mode: *p* = 0.012) resulting from the combination of these baseline indices. B) The volcano plot was constructed based on the 197 differential plasma metabolites between craniopharyngioma (CP, n = 52) and healthy controls (HC, n = 50), with each point representing a metabolite. Red indicates increased metabolites, while blue represents the 137 decreased metabolites in the plasma of CP patients. C) Bar plots display the enriched chemical classes based on differential metabolites, with color depth and bar length representing the p‐value and enrichment ratio, respectively. D) The significantly differential metabolites in patients with CP (blue) and HC (orange), stratified by average concentration. Only the 20 most abundant metabolites are shown. E) The bubble plot illustrates the enriched metabolic pathways based on differential metabolites, with color depth and bubble size indicating the p‐value and enrichment ratio, respectively. F) Bagplots illustrate the potential origins of differential metabolites and the corresponding number of metabolites from each source. G,H) Scatter plots generated by MIMOSA2 depict the linear relationship between the concentration of hypoxanthine and the microbial community metabolic potential (CMP) resulting from the three contributing genes (K03783, K03784, and K10213). I) A random forest model based on the combination of gut metagenomics and serum metabolomics was constructed, selecting the eight metabolites/species with a higher relative contribution to the model's accuracy. J) The ROC curve presents the efficacy of the random forest model, with an AUC of 0.979 (95% CI: 0.942–1). **p < 0.01.

The separation between the CP and HC groups was evident in unsupervised principal components analysis (PCA) and supervised orthogonal partial least squares‐discriminant analysis (OPLS‐DA), as depicted in the figures (Figure S5A–D, Supporting Information). OPLS‐DA mode provided an S‐plot to identify the metabolites contributing to group separation (Figure S5E–F, Supporting Information). We also conducted RDA for both positive and negative modes, revealing a significant effect from the combination of these baseline indices (positive mode: *p* = 0.006; negative mode: *p* = 0.012) (**Figure** **3**A). Further examination of individual indices highlighted the significant association of BMI (positive mode: R^2^ = 0.191, *p* = 0.001; negative mode: R^2^ = 0.285, *p* = 0.001) with the grouping effect.

In total, we identified 197 metabolic substances with variable importance projection (VIP) values >1, *p* values (ANOVA) <0.05, and max fold change value ≥1, with 60 metabolites upregulated and 137 metabolites downregulated in the CP group (Table S8, Supporting Information; Figure 3B). After normalizing by BMI, we categorized the differential metabolites into various chemical classes by aligning them with PubChem (Figure 3C; Figure S5G, Supporting Information). And differential chemical classes included organic acids (increased in HC), fatty acyls (increased in HC), and lipids and lipid‐like molecules (increased in HC). Notably, specific metabolites, such as 1,1′−[1,11−Undecanediylbis(oxy)] bisbenzene, adlupulone, and hypoxanthine, were enriched in the CP group, while 4‐ene‐valproic acid, cis‐4‐hydroxycyclohexylacetic acid, and 2‐hydroxy‐2,6,6‐trimethylcyclohexanone were significantly more enriched in the HC group (Figure 3D).

Pathway enrichment analysis revealed alterations in purine metabolism (upregulated in CP), steroid hormone biosynthesis (downregulated in CP), biosynthesis of unsaturated fatty acids (upregulated in CP), and drug metabolism‐cytochrome P450 (upregulated in CP) (Figure 3E), with hypoxanthine, 2‐methoxyestrone 3‐glucuronide, eicosapentaenoic acid, and 3‐hydroxylidocaine identified as associated pathways (Figure S5H, Supporting Information).

### 2.5 Integrative Analysis of Gut Microbiome and Serum Metabolome in CP

We further explored potential relationships between gut microbiota and serum metabolites. Annotation to MetOrigin revealed that the sources of the 197 differential metabolites could be classified as host (n = 2), microbiota (n = 8), host‐microbiota co‐metabolism (n = 4), and others with heterogeneous origins (n = 183) (Figure 3F).

To delve deeper into the interaction between serum metabolites and the gut microbiota, we employed MIMOSA2 to uncover the contribution of the microbiome to metabolites. Among the differentially expressed metabolites, hypoxanthine was significantly influenced by the gut metagenome (R^2^ = 0.015, *p* = 0.027). This microbial community metabolic potential was attributed to K03783 (purine nucleoside phosphorylase [EC:2.4.2.1]), K03784 (purine nucleoside phosphorylase [EC:2.4.2.1]), and K10213 (ribosylpyrimidine nucleosidase [EC:3.2.2.8]), all of which were notably higher in patients with CP (Figure 3G,H). Subsequently, we examined the biochemical reactions involving the three contributing genes through MetOrigin analysis and found that R01863 (inosine + orthophosphate ≤> hypoxanthine + alpha‐D‐ribose 1‐phosphate) and R02748 (deoxyinosine + orthophosphate ≤> hypoxanthine + 2‐deoxy‐D‐ribose 1‐phosphate) might serve as the bridges between the altered gut microbiome and aberrant serum metabolome in CP (Figure S5I, Supporting Information).

Subsequently, we constructed machine learning models based on the gut microbiome and serum metabolome in order to testify their potential as biomarkers of CP. Integrating gut metagenomics and serum metabolomics, we conducted a tenfold cross‐validation on the random forest model to validate the fidelity of the results. Hypoxanthine, 2‐Methoxyestrone 3‐glucuronide, *Ruminococcus torques*, and *Clostridium sp CAG 7* were detected as the components with higher contribution to the accuracy of the model (Figure 3I). And this model reached an AUC value of 0.979 (95% CI: 0.942∼1), indicating that gut microbiome and serum metabolome might serve as potent biomarkers of CP (Figure 3J).

### 2.6 Construction of CP Animal Model and Validation of Clinical Resemblance

**Figure 4:**
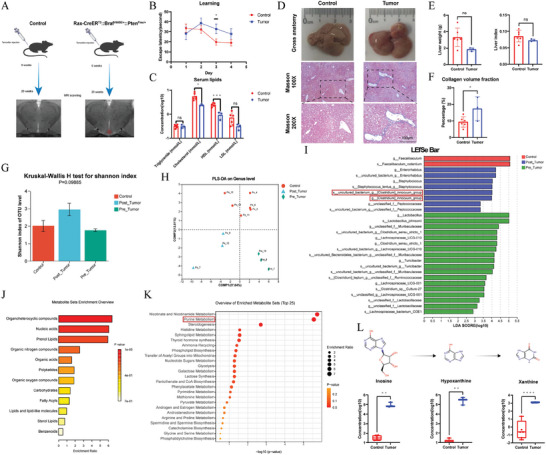
Phenotypic analyses and multi‐omics investigation in transgenic rodent model with CP reaffirmed the hypothalamic comorbidities related signatures. A) The schematic diagram illustrates the time span from tamoxifen induction (tumor induction) to MRI scan (tumor confirmation), which lasted for 20 weeks. B) The line chart of the water maze demonstrates that mice with craniopharyngioma (tumor group, n = 3) exhibited a significantly prolonged escape latency compared to their wild‐type littermates (control group, n = 6) on the third day of the learning phase (32.80 ± 4.93 vs 19.73 ± 3.04). C) Bar charts of serum lipids display significantly decreased levels of cholesterol (2.40 ± 0.12 vs 5.53 ± 0.75) and HDL (0.92 ± 0.18 vs 2.45 ± 0.16). D) Gross anatomy and histopathological analysis (Masson staining) indicate intensified fibrosis in the rodent model with CP E) with no significant differences in liver weight or liver index. F) Further quantitative analysis of histopathological results reveals a notable increase in collagen volume fraction in mice with CP (17.32 ± 4.15 vs 9.35 ± 1.26). G) The bar chart displays an increased Shannon index in the post‐tumor‐formation rodent model compared to the wild‐type control and pre‐tumor‐formation rodent model (*p* = 0.09885). H) PLS‐DA on the genus level depicts clear separation among the post‐tumor‐formation rodent model, wild‐type control, and pre‐tumor‐formation rodent model, with PERMANOVA indicating significantly altered beta diversity (R^2^ = 0.584, *p* = 0.002). I) LEfSe analysis reveals that the post‐tumor‐formation rodent model is featured with an increased genus *Clostridium innocuum* group. J) Enrichment analysis of serum metabolomics finds that the differential metabolites are mainly classified as organoheterocyclic compounds, nucleic acids, and prenol lipids. K) Pathway enrichment analysis of serum metabolomics indicates that purine metabolism is significantly altered between the post‐tumor‐formation rodent model and wild‐type control. L) Among the metabolites annotated to purine metabolism, hypoxanthine and its upstream metabolite (inosine) as well as downstream metabolite (xanthine) are significantly enriched in the tumor group. *p < 0.05, **p < 0.01, ***p < 0.001, ****p < 0.0001.

To further confirm the utility of gut microbiome and serum metabolome as biomolecular signatures for CP, we employed a recently developed transgenic murine model of papillary CP. After tamoxifen induction, we subjected the animal models (Rax‑CreER^T2^::Braf^V600E/+^::Pten^Flox/+^) and their wild‐type littermates to a Western diet (WD) to replicate modern dietary habits. Following 20 consecutive weeks, we confirmed the presence of intracranial lesions in the enrolled animals using MRI (**Figure** **4**A).

To assess if the animal models exhibited neurocognitive characteristics similar to patients, we conducted the Morris water maze test. Mice with CP (tumor group) demonstrated a significantly prolonged escape latency compared to their wild‐type littermates (control group) on the third day of the learning phase (Figure 4B). However, there were no significant differences in the parameters of the memory phase between the two groups (Figure S6A–C, Supporting Information). Upon euthanasia, we prepared serum samples for biochemical analyses, revealing significantly lower HDL and cholesterol levels in the tumor group (Figure 4C). Notably, liver and renal functions showed no significant alterations between the two groups (Figure S6D,E, Supporting Information). Additionally, we measured body and liver weights and conducted Masson's trichrome staining on liver samples to investigate liver injuries in animal models. We observed enhanced collagen deposition in the histopathological study (Figure 4D,F), although there were no significant changes in liver weight or liver index (Figure 4E). In summary, behavioral experiments, serum biochemical data, and liver histopathological analyses collectively indicated that the tumor group exhibited impaired neurocognition, disrupted lipid profiles, and liver injury, mirroring the clinical features of patients with CP (neurocognitive impairment and liver injury).

### 2.7 Validation of Gut Microbial and Serum Metabolomic Fingerprint in CP Animal Model

To further explore the parallels between the characteristic gut microbiome and serum metabolome in CP, we conducted fecal 16S rRNA sequencing and serum metabolomics in a CP animal model. Compared with the wild‐type control and pre‐tumor formation rodent models, the post‐tumor formation rodent model exhibited an increased Shannon index (*p* = 0.09885, Figure 4G) and significant alterations in beta diversity (R^2^ = 0.584, *p* = 0.002, Figure 4H), consistent with findings in our clinical cohort. Regarding differential taxa among the three groups, ternary analysis indicated that the pre‐tumor formation rodent model and wild‐type control were characterized by the genera *Lactobacillus* and *Faecalibaculum*, respectively (Figure S6F, Supporting Information). Subsequent LEfSe analysis (Figure 4I), along with the Kruskal–Wallis test (Figure S6G, Supporting Information), revealed that the post‐tumor formation rodent model featured an increased abundance of *Clostridium innocuum*, similar to the taxonomic alterations found in patients with CP.

Regarding serum metabolomic findings, enrichment analysis suggested that the differential metabolites were mainly classified as organoheterocyclic compounds, nucleic acids, and prenol lipids (Figure 4J). Consistent with results from our clinical cohort, pathway enrichment analysis in the murine model indicated significant alterations in purine metabolism between the post‐tumor formation rodent model and the wild‐type control (Figure 4K). Among the metabolites annotated for purine metabolism, hypoxanthine, its upstream metabolite (inosine), and its downstream metabolite (xanthine) were significantly enriched in the tumor group (Figure 4L). Considering the cognitive and metabolic phenotypes observed in the post‐tumor formation rodent model, the increased abundance of the genus *Clostridium* and perturbed purine metabolism may play roles in the development of impaired neurocognition and liver injury in a rodent model of CP.

### 2.8 Perturbed Purine Metabolism and Increased *Clostridium* Might Facilitate the Development of Hypothalamic Syndrome Through Dysregulated Anti‐Oxidative System

To exclude the potential bias from metabolic disorders, we have shortened the timespan of WD intervention and added the chow diet (CD) group as control. To summarize, we have established four groups (Control + CD, Tumor + CD, Control + WD, Tumor + WD), and the duration of diet intervention of all groups was set at 8 weeks. There are no significant alterations in collagen volume, oil‐red positive area (Figure S7A,B, Supporting Information), and performance in water maze experiment (Figure S7C,D, Supporting Information) between control group and tumor group under chow diet. However, after 8‐week exposure to the western diet, significant elevation of collagen volume as well as oil‐red positive area (Figure S7A,B, Supporting Information), and notable worsening of performance in water maze experiment (Figure S7C,D, Supporting Information) were observed in tumor group compared with control group. These results indicated that CP itself might result in metabolic and cognitive vulnerability compared with control group when under exposure to external challenge such as western diet.

To explore the roles of purine metabolism and microbiota in the metabolic and cognitive vulnerability of CP, we then inspected the alteration in purine metabolism‐related genes of targeted organs (liver and cortex) and gut microbiome in those under western diet intervention. We found that genetic expression of crucial enzymes in purine metabolism (Hypoxanthine‐guanine phosphoribosyl transferase, HPRT, Xanthine oxidoreductase, XOR, and Purine nucleoside phosphorylase, PNP) were significantly increased in Tumor + WD group. Also, since purine metabolism is closely associated with the maintenance of redox homeostasis, we examined the genetic expression of well‐known biomarkers of anti‐oxidative system, including nuclear factor erythroid 2–related factor 2 (Nrf2), superoxide dismutase 1 (SOD‐1) and Catalase (CAT). The results revealed that a notable increase in Tumor + WD group (Figure S7E,F, Supporting Information). Regarding alteration in gut microbiome, there are no significant alterations in Shannon index and beta diversity between Control + WD group and Tumor + WD group (Figure S7G,H, Supporting Information). However, the relative abundance of genus *Clostridium sensu stricto 1* was significantly higher in Tumor + WD group (Figure S7I, Supporting Information). Taken together, these results indicated that perturbed purine metabolism, abnormally activated anti‐oxidative system, and increased *Clostridium* might play a role in the metabolic and cognitive vulnerability of CP.

**Figure 5:**
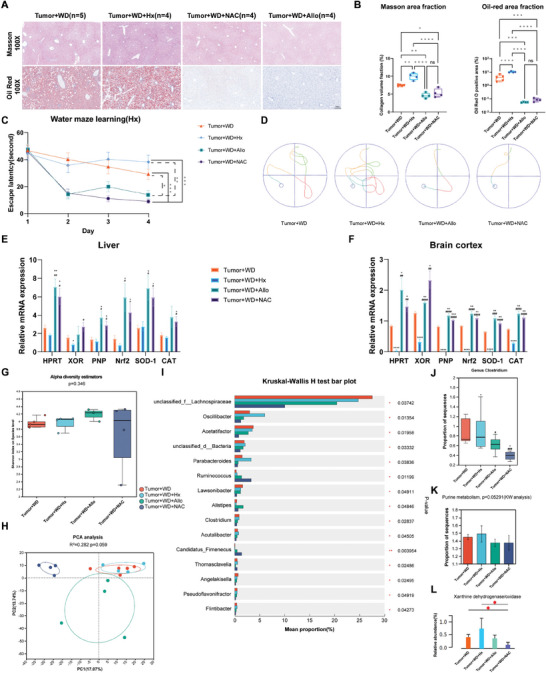
Intervention experiments in CP mouse model revealed that dysregulated purine metabolism, increased Clostridium, and redox imbalance might be involved in the development and progression of hypothalamic comorbidities in CP. A) The histopathological analyses (Masson staining and Oil red O staining) indicate intensified fibrosis and elevated lipid accumulation in the CP mice treated with WD + Hx. B) Quantitative analyses of histopathological results reveal a notable increase in collagen volume fraction (9.95 ± 0.50) and oil red area fraction (10.06 ± 0.64) in CP mice treated with WD + Hx, meanwhile NAC (collagen volume fraction: 5.29 ± 0.60; oil red area fraction: 0.08 ± 0.01) and Allo treatment (collagen volume fraction: 4.63 ± 0.36; oil red area fraction: 0.06 ± 0.001) induced remarkable alleviation in parameters above. C) The line chart of the water maze demonstrates that CP mice in WD + Hx group exhibited a significantly prolonged escape latency (38.28 ± 5.22) on the last day of the learning phase, while NAC (8.91 ± 1.42) and Allo (13.90 ± 2.95) treatment brought about remarkable decrease of this parameter. D) The representative trajectory diagram of each group in the water maze experiment. E,F) In the target organs (liver and brain cortex), Hx treatment suppressed the relative mRNA expression of critical enzymes in purine metabolism (HPRT, XOR, and PNP) as well as crucial players in anti‐oxidative system (Nrf2, SOD‐1, and CAT), meanwhile, NAC and Allo treatment induced significant upregulation in relative expression of these genes. G) There is no remarkable deviation in Shannon index among different groups. H) PCA analysis found separation in beta diversity between groups (WD + NAC and WD + Allo) with alleviated hypothalamic syndrome and their counterparts (WD and WD + Hx). I) Kruskal–Wallis test demonstrated that genus *Clostridium* notably varied among different treatment groups and co‐variated with the severity of hypothalamic syndrome. J) Specifically, Hx treatment induced enrichment of genus *Clostridium* (0.92 ± 0.49), while both NAC (0.39 ± 0.10) and Allo (0.62 ± 0.19) treatment resulted in depletion of genus *Clostridium*. K) Hx treatment tended to increase the proportion of genes related to purine metabolism, while both NAC and Allo treatment were prone to result in diminishment of this pathway. L) Hx treatment induced a remarkable increase in relative abundance of XOR (xanthine dehydrogenase/oxidase), while NAC treatment significantly reduced the genetic expression of this enzyme. CP: craniopharyngioma; WD: western diet; Hx: hypoxanthine; NAC: N‐Acetyl Cysteine; Allo: allopurinol; HPRT: Hypoxanthine‐guanine phosphoribosyl transferase; XOR: Xanthine oxidoreductase; PNP: Purine nucleoside phosphorylase; Nrf2: Nuclear factor erythroid 2‐related factor 2; SOD‐1: Superoxide dismutase 1; CAT: Catalase. *p < 0.05, **p < 0.01, ***p < 0.001, ****p < 0.0001; #*p* < 0.05, ##*p* < 0.01, ###*p* < 0.001, ####*p* < 0.0001; in figure E, F and J, * compared with Tumor + WD group; # compared with Tumor + WD + Hx group. N = 5 in CP+WD group, n = 4 in CP+WD+Hx group, n = 4 in CP+WD+NAC group, n = 4 in CP+WD+Allo group.

To inspect the potential influence on CP‐related hypothalamic comorbidities from perturbed purine metabolism, we treated CP mouse model with hypoxanthine (Hx), N‐Acetyl Cysteine (NAC), and allopurinol (Allo). Masson staining across different groups revealed that Hx treatment significantly upregulated the collagen volume fraction, while NAC and Allo treatment brought about remarkable decrease in collagen volume fraction. Regarding oil‐red staining, similar trends were observed in the quantitative measurement of oil‐red O positive area (**Figure** **5**A,B). With regards to cognitive performance, the water maze experiment found that Hx notably prolonged the escape latency, while NAC and Allo treatment significantly shortened the escape latency (Figure 5C,D).

In order to explore the underlying molecular mechanism of CP‐related liver injury and cognitive impairment, we performed qPCR analysis in liver and cortex samples of CP mouse model. As a result, Hx treatment suppressed the relative mRNA expression of critical enzymes in purine metabolism (HPRT, XOR, and PNP) as well as crucial players in anti‐oxidative systems (Nrf2, SOD‐1, and CAT), meanwhile, NAC and Allo treatment induced significant upregulation in genetic expression of targets mentioned above (Figure 5E,F). Also, we inspected the gut microbial alterations among different treatment groups. Although there is no remarkable deviation in Shannon index among different groups (Figure 5G), PCA analysis detected separation in beta diversity between groups (WD + NAC and WD + Allo) with alleviated hypothalamic syndrome and their counterparts (WD and WD + Hx) (Figure 5H). Subsequently, we conducted Kruskal–Wallis test to explore characteristic taxa correlating with severity of hypothalamic syndrome in CP mouse model. In line with the results observed in clinical cohort and pilot animal study, we found that genus *Clostridium* notably varied among different treatment groups and co‐variated with the severity of hypothalamic syndrome. Specifically, Hx treatment induced enrichment of genus *Clostridium*, while both NAC and Allo treatment resulted in depletion of genus *Clostridium* (Figure 5I,J). Further, we examined the alteration in purine metabolic capacity of gut microbiota, and we found that Hx treatment tended to increase the proportion of genes related to purine metabolism, while both NAC and Allo treatment were prone to result in diminishment of this parameter (Figure 5I,J). Specifically, Hx treatment induced a remarkable increase in relative abundance of XOR (xanthine dehydrogenase/oxidase), while NAC treatment significantly reduced the genetic expression of this enzyme (Figure 5K,L). Together, these results indicated that excessive hypoxanthine could result in increased *Clostridium* along with microbial purine metabolic capacity, and this might further induce suppressed purine metabolism as well as malfunctioned anti‐oxidative system in target organs (liver and brain cortex). The interaction among them might mediate the development and progression of hypothalamic comorbidities (liver injury and cognitive impairment) in CP.

In summary, through integrating clinical cohort and animal model of CP, increased genus *Clostridium* and perturbed purine metabolism emerge as potential biochemical fingerprints with pathophysiological implications for CP‐associated hypothalamic comorbidities.

## 3 Discussion

This study represents the first comprehensive profiling of the gut microbiome and serum metabolome in both patients and rodent models of CP. In our clinical cohort, we identified significant enrichments of the genera *Clostridium*, *Eubacterium*, and *Roseburia* in patients with CP, particularly those with higher degrees of hypothalamic involvement. Correlation analysis further revealed positive associations between CP‐characteristic taxa and the development of hypothalamic comorbidities (dyslipidemia, cognitive impairment, and liver injury). Additionally, our serum metabolomic assessment unveiled perturbed purine metabolism and elevated levels of hypoxanthine in CP group. Subsequent integrative analyses revealed potential interactions between CP‐specific microbiome and serum hypoxanthine levels, and demonstrated their potential as vital biomarkers of CP. In the further animal study, we confirmed that increased *Clostridium* and perturbed purine metabolism served as shared biomarkers between clinical patients and transgenic rodent models of CP. Also, increased *Clostridium* and perturbed purine metabolism were also found to be associated with the development of hypothalamic comorbidities in mouse model. Finally, we performed different treatments targeting hypoxanthine, and found that hypoxanthine excess could exacerbate hypothalamic comorbidities while pharmacological interventions (NAC or Allo) to eliminate hypoxanthine could alleviate hypothalamic comorbidities. Mechanistically, hypoxanthine co‐variated with the abundance of *Clostridium*, and its influence on hypothalamic comorbidities might be mediated by crucial enzymes involved in purine metabolism (HPRT, XOR, and PNP) and anti‐oxidative system (Nrf2, SOD‐1, and CAT).

The gut microbiome of patients with heightened hypothalamic involvement was characterized by an increased abundance of the genera *Eubacterium*, *Clostridium*, *Roseburia*, and their subordinate species, which have previously been reported to be increased in obese individuals.^[^ ^22^ ^]^ Correlation analyses revealed that *Eubacterium rectale* and *Roseburia inulinivorans* were positively associated with serum lipid levels (cholesterol and sdLDL), while genus *Clostridium* and *Clostridium sp. CAG 7* were positively correlated with neurocognitive dysfunction. Previous research has identified *Clostridium* species were altered in anorexia nervosa and positively correlated with estimates of mental disorder, indicating a potential role of these species in the regulation of eating behavior and neuropsychiatric symptoms. This effect could be partially due to the tryptophanase encoded by *Clostridium*, which is the critical enzyme converting tryptophan to indole, pyruvate, and ammonia.^[^ ^23^ ^]^ Moreover, Misiak et al. discovered that patients with schizophrenia had higher *Eubacterium fissicatena* and *Clostridium innocuum*.^[^ ^24^ ^]^ Huang et al. speculated that the flourish of *Eubacterium rectale* resulted in a reduction in short‐chain fatty acid production, which explains the cognitive dysfunction in diabetes.^[^ ^25^ ^]^ Moreover, *Clostridium* was regarded as the microbiota associated with inflammation and subsequently resulted in dyslipidemia and liver injury.^[^ ^26^ ^]^

Subsequent serum metabolomic and integrative analyses unveiled the potential influence of the gut microbiome on serum hypoxanthine levels through participation in purine metabolism‐related biological reactions. Hypoxanthine, an intermediate in the purine degradation pathway, is subject to regulation by both host and gut microbial metabolism.^[^ ^27^ ^]^ Prior research has linked hypoxanthine to various pathological conditions, including obesity, metabolic syndrome,^[^ ^28^ ^]^ NAFLD,^[^ ^29^ ^]^ neuroinflammatory status,^[^ ^30^ ^]^ neuroenergetic abnormalities, and fatigue.^[^ ^31^ ^]^ In the context of this study, MIMOSA2 analysis suggests that an altered gut microbiome may contribute to elevated serum hypoxanthine levels in patients with CP. Further metabolomic analysis in rodent models of CP confirmed the perturbations of purine metabolism and elevation of serum hypoxanthine. Given that individuals with CP are predisposed to developing metabolic syndrome, NAFLD, impaired cognition, and fatigue, we speculate that gut microbiome‐derived hypoxanthine may play a role in the development of these pathophysiological conditions.^[^ ^19^, ^32^ ^]^

The hypothalamic comorbidities have major adverse effects on long‐term prognosis and QoL of CP, while the biomarkers and underlying mechanism remained elusive.^[^ ^33^ ^]^ In the current study, we revealed that gut microbiome and serum metabolome are closely associated with hypothalamic comorbidities of CP and they could concomitantly serve as potent biomarker of CP. Our findings might shed light on the early detection and prophylactic intervention for CP patients, especially those at risk of developing hypothalamic comorbidities. Moreover, these findings indicate the possibility that gut microbiome‐associated hypoxanthine might be involved in the development and progression of hypothalamic comorbidities of CP.

To test our hypothesis, we conducted different treatments targeting hypoxanthine. Since high concentrations of hypoxanthine may interfere with the purinergic signaling, we first inspected the relative genetic abundance of pivotal enzymes in purine metabolism, and the results indicated that the influence of hypoxanthine on hypothalamic syndrome in CP is closely associated with a decrease in purinergic enzymes (HPRT, XOR, and PNP). The lack of HPRT, the enzyme catalyzing the salvage pathway of hypoxanthine, has been reported to be associated with neurological abnormalities. XOR, the enzyme degrading hypoxanthine, exists in two forms that are derived from a single gene (XDH).^[^ ^34^ ^]^ The reduced form of XOR is referred to as xanthine dehydrogenase (XDH), and the oxidized form as xanthine oxidase (XO). XDH can be post‐translationally modified to XO via proteolysis or oxidation of critical cysteines.^[^ ^35^ ^]^ The XDH form has greater abundance and affinity for NAD^+^ as the electron acceptor to generate NADH, while the XO form is mainly associated with the production of large amounts of O_2_^−^ and H_2_O_2_ by preferentially using oxygen as the electron acceptor.^[^ ^36^ ^]^ Under healthy conditions, XDH is constitutively expressed, and XO levels are low.^[^ ^37^ ^]^ However, XDH conversion into XO is favored by pathological conditions including hypoxia, low pH, ischemia, inflammation, and the presence H_2_O_2_ itself,^[^ ^38^ ^]^ and it is extremely difficult to know the relative concentration of the two forms in living organisms and cells.^[^ ^39^ ^]^ The final product of purine catabolism is uric acid, an antioxidant capable of protecting cells and organs from reactive oxyradicals. Apart from being a potent antioxidant, uric acid has been claimed as a stimulator of the cerebral cortex, and low uric acid concentrations have been associated with neurodegenerative diseases.^[^ ^40^ ^]^ Besides, alterations of purine metabolism have been reported to be involved in the pathogenesis of the metabolic syndrome and insulin resistance.^[^ ^40^, ^41^ ^]^ In the current study, hypoxanthine treatment of CP mouse model resulted in exacerbated hypothalamic syndrome manifestations and parallel suppression of pivotal purinergic enzymes in corresponding organs, indicating that alterations of products in purine metabolism (such as uric acid and reactive oxygen species, ROS) might be involved in the development and progression of hypothalamic syndrome in CP.

Given that both manifestations of hypothalamic syndrome and purine metabolism have been associated with redox imbalance respectively, we next examined the relative abundance of crucial players in anti‐oxidative system (Nrf2, SOD‐1, and CAT). Nuclear factor erythroid 2–related factor 2 (Nrf2) is a redox‐sensitive transcription factor that provides cyto‐protection against oxidative stress.^[^ ^42^ ^]^ Under oxidative stress, Nrf2 dissociates from Keap1 protein and trans‐locates to the nucleus, eventually promoting the expression of genes encoding antioxidant and phase 2 detoxifying enzymes like SOD1 and CAT.^[^ ^43^ ^]^ However, when ROS overwhelms the intrinsic cellular antioxidant system, either via an abnormal overproduction of ROS or reduction of their antioxidant capacity, they contribute to pathogenesis (oxidative stress), causing transient or permanent damage to nucleic acids, proteins, and lipids.^[^ ^44^ ^]^ In the current study, we demonstrated that both Allo (a specific inhibitor of XO activity^[^ ^45^ ^]^ with parallel ROS lowering effect as a direct ROS scavenging moiety^[^ ^46^ ^]^) and NAC (a non‐specific ROS scavenger) could alleviate the hypothalamic syndrome in CP mouse model, concomitantly reversing the suppressed purine metabolism and diminished anti‐oxidative system. Taken together, our results imply that redox imbalance might be involved in the influence of dysregulated purine metabolism on hypothalamic syndrome in CP.

Indeed, intricate interactions were reported between purine metabolism and anti‐oxidative system. On the one hand, Nrf2–HK2 complex was found to be indispensable for XOR expression.^[^ ^47^ ^]^ On the other hand, XOR promotes USP15‐mediated Nrf2‐KEAP1 signaling and subsequently reactive oxygen species production.^[^ ^48^ ^]^ Besides, uric acid administration resulted in significant neuroprotection for dopaminergic neurons probably through Nrf2‐ARE‐induced inhibition of oxidative damage and neuroinflammation in a Parkinson's disease mouse model.^[^ ^49^ ^]^ However, the precise underlying mechanisms of interactions among altered gut microbiome, accumulated hypoxanthine, redox imbalance, and CP‐related hypothalamic syndrome warrant further study.

The clinical section of this study does have certain limitations, including a relatively small sample size with one ethnicity and a lack of data on potential confounding factors, such as dietary patterns and feeding rates. Consequently, the results should be interpreted with caution, and further studies with larger sample size, different ethnicities, and comprehensive data are warranted. In the realm of animal studies, this investigation serves as a preliminary pilot exploration, and additional experimental validation is warranted to confirm the roles of the gut microbiota and related microbial metabolites. While we have identified candidate gut microbiota and serum metabolites, their specific contributions to CP ‐associated hypothalamic comorbidities and corresponding mechanisms remain under‐explored. Further research is needed to validate our findings and delve into the interactions between gut microbiota, serum metabolite, CP‐associated hypothalamic comorbidities, and even CP itself. Last but not least, the technologies (16S rRNA sequencing, metagenomics, and untargeted metabolomics) employed in this study both exhibit certain limitations. For example, 16S rRNA sequencing mainly targets the 16S rRNA region of microbiota, which is limited and highly conserved, while metagenomic sequencing provides higher resolution and more comprehensive information by sequencing the entire microbial community DNA. Therefore, future studies might take advantage of technology with potential merits, such as fecal proteomics and quantitative metabolomics.

In conclusion, our study revealed that increased *Clostridium* and perturbed purine metabolism are shared between patients and rodent models of CP. Further intervention experiments indicated that interactions among elevated *Clostridium*, dysregulated purine metabolism, and redox imbalance might mediate the development and progression of CP‐associated hypothalamic comorbidities. We hope that these preliminary findings will spark further inquiries into this field, ultimately fostering advances in CP prevention, diagnosis, and treatment.

## 4 Experimental Section

### Participants

This study received the approval from the Ethics Committee of Huashan Hospital (KY2015‐256) at Fudan University, with informed consent from all participants. Patients diagnosed with CP at Huashan Hospital, Department of Neurosurgery, between March 2018 and April 2019 were recruited. Diagnosis of CP was established through standard pathological analysis.^[^ ^50^ ^]^ Exclusion criteria encompassed: 1) antibiotic and/or probiotic usage within the preceding 6 months, 2) a history of gastrointestinal tumors and/or inflammatory diseases, and 3) immunosuppressive drug usage within the past 6 months. Healthy controls (HC) selected for 16S rRNA sequencing, matched for general demographics, were drawn from a sizable cohort established by the Department of General Surgery at Shanghai Tenth People's Hospital, School of Medicine, Tongji University. HC for metagenomic sequencing was selected from a sizable cohort established by the First Affiliated Hospital of Chongqing Medical University.^[^ ^51^ ^]^ The exclusion criteria for HC mirrored those applied to patients with CP.

### 16S rRNA Gene Sequencing

16S rRNA gene sequencing was executed in accordance with previously established procedures.^[^ ^52^ ^]^ Briefly, the V3–V4 region of the bacterial 16S ribosomal RNA gene underwent PCR amplification. Amplicons were extracted from 2% agarose gels and purified via the AxyPrep DNA Gel Extraction Kit. Quantified amplicons were pooled to equalize their concentrations for sequencing via an Illumina MiSeq (Illumina, Inc., CA, USA). Paired‐end reads of 250 bp were overlapped on their 3′ ends to concatenate into original longer tags using PANDAseq (https://github.com/neufeld/pandaseq, version 2.9). Operational taxonomic units (OTUs) profiling tables and alpha diversity analyses were conducted using Python scripts from QIIME. Weighted UniFrac, which incorporates phylogenetic information to compare species community differences among samples, was employed in this study, with the original script available on the QIIME website (http://qiime.org/scripts/beta_diversity.html). Clustering UniFrac results grouped samples with similar beta diversity. The outcomes were presented through the analysis of similarities (ANOSIM) and Adonis analysis, with Adonis results further visualized through principal coordinate analysis. To evaluate the impact of basic information, redundancy analysis (RDA) and permutation tests were carried out using GenesCloud tools (https://www.genescloud.cn), generating p‐values for the permutation test and parameters (R^2^ and p‐value) for individual indices. Subsequently, the Microbiome Multivariate Association with Linear Models (MaAsLin2) in R was employed to adjust for significant confounding factors identified in the RDA.^[^ ^53^ ^]^ The rank‐sum test was applied to analyze significant differential taxa between different groups. Specifically, the Wilcoxon test function from the stats package in R was used for the difference analysis between two groups, while the Kruskal test function from the same package in R was utilized for the difference analysis involving more than two groups.

### Shotgun Metagenomic Sequencing

Shotgun metagenomic sequencing was executed following the established protocol as previously described.^[^ ^52^ ^]^ The process involved the construction of DNA libraries. Raw Illumina reads were screened as detailed in the previous publication. The preprocessing of reads included assembly using SOAPdenovo software (version 1.05).^[^ ^54^ ^]^ The k‐mer with the highest N50 value was selected for the final assembly. Prediction of open reading frames (ORFs) in the assembled scaffolds was conducted using MetaGeneMark software (http://exon.gatech.edu/GeneMark/metagenome/Prediction/).^[^ ^55^ ^]^ A non‐redundant gene catalog was created through a pairwise comparison of the predicted ORFs (gene length > 100 bp) using CD‐HIT (version 4.5.7).^[^ ^56^ ^]^ Clean reads were aligned to the genes in the non‐redundant catalog using SOAPaligner. Gene abundance calculations followed the formula from Qin et al.^[^ ^57^ ^]^ To annotate non‐redundant genes, BLAT (v35)^[^ ^58^ ^]^ was employed, comparing assembled protein sequences to the Kyoto Encyclopedia of Genes and Genomes (KEGG) database. When the similarity between the assembled protein sequence and a protein sequence in the database was deemed significant (minScore = 60 and E value < 1e − 5), the assembled protein was considered to fulfill the same role as the database protein. The relative abundance of all orthologous genes was computed to determine the relative abundance of each KO. Shotgun metagenomic sequencing was conducted following the previously outlined procedure.^[^ ^52^ ^]^ Based on sample abundance, the Bray–Curtis distance between samples was calculated, followed by hierarchical clustering to assess the distance relationships between samples and the presence of outliers. ANOSIM and Adonis were performed for validation. Subsequent RDA and MaAsLin2 analyses were carried out as described earlier for 16S rRNA sequencing. The Wilcoxon test function from the stats package in R was employed for analyzing differences between two groups, while the Kruskal test function from the same package in R was used for analyzing differences among more than two groups. To further explore potential internal relationships, Spearman correlations between the abundances of differential taxa and clinical parameters of the patients were conducted. The results were presented through scatter plots or heat maps generated using GraphPad 8.0.

### Untargeted HPLC‐MS Metabolomics Measurement of Metabolite Labeling and Concentration

The collection and processing of fasting plasma samples were conducted as previously described.^[^ ^59^ ^]^ These samples were subjected to high‐resolution mass spectrometry analysis. Metabolite separation was achieved through gradient elution, following established procedures. Data acquisition and processing were performed using UNIFI version 1.8.1. Raw data from the metabolic profiling experiments underwent processing with Progenesis QI v2.3 data analysis software, encompassing peak picking, alignment, and normalization, resulting in peak intensities for retention time (Rt) and m/z data pairs. Compound identification was carried out using the HMDB and LipidMaps Database v2.3. Subsequent statistical analysis utilized normalized peak intensities through EZin for V3.0.3. To discern prominent differences between groups, metabolic compound data underwent analysis employing principal component analysis (PCA) and orthogonal partial least square‐discriminant analysis (OPLS‐DA). The key parameters for identifying variables contributing to classification included variable importance projection (VIP) generated by OPLS‐DA and maximum fold change. Variables meeting the criteria of VIP values >1, *p* values (ANOVA) < 0.05, and max fold change value ≥ 1 were selected as potential significant differential metabolites. RDA was performed to assess the potential influence of basic information, as described in the 16S rRNA sequencing section. Subsequent normalization and enrichment analyses, considering chemical class and pathway, were carried out using MetaboAnalyst 5.0 (www.Metaboanalyst.ca).^[^ ^60^ ^]^

### Integrative Analysis of Gut Metagenomics and Serum Metabolomics

The integration of gut metagenomics and serum metabolomics was achieved through MetOrigin^[^ ^61^ ^]^ and MIMOSA2.^[^ ^62^ ^]^ For machine learning modeling, the random forest package in R was utilized, incorporating both gut metagenomics and serum metabolomics data. The data were divided into training and test sets (training set: validation set = 7:3), and a machine learning model was generated through tenfold cross‐validation within the training set, followed by testification of efficacy in the test set. The random Forest package in R was used for this analysis.

### Animal Model

All experiments described in this study received approval from the Animal Care and Use Committee of Fudan University (2022JS‐339) and adhered to the Regulations for Laboratory Animal Management by the Ministry of Science and Technology of the People's Republic of China. The animals used in this study were graciously provided by Professor Qingfeng Wu^[^ ^63^ ^]^ and were housed in controlled environmental conditions, including a room temperature of 20–22 °C, 50% humidity, and a 12 h light/dark cycle. They were provided with free access to water and a Chow diet/Western diet.

### Animal Treatments

For chronic hypoxanthine treatment experiments, CP mice were given ad libitum access to a Western diet with or without Hx (20 mg g^−1^) (Sango Biotech, China, purity ≥ 98.0%) and water. For the allopurinol treatment experiment, CP mice were maintained on 2 g L^−1^ allopurinol (Med Chem Express) in the drinking water and a Western diet for 8 weeks. For N‐acetylcysteine treatment experiment, CP mice were supplemented with 150 mg L^−1^ N‐acetylcysteine (Sango Biotech, China) in drinking water and a Western diet for 8 weeks.

### Statistical Analysis

Clinical metadata underwent statistical analysis using SPSS software (version 16.0; IBM Corporation, Armonk, NY, USA). Continuous data were presented as mean ± standard deviation (interquartile range for non‐normalized data), while count data were expressed as counts and proportions. T‐tests were utilized to assess differences between normally distributed continuous variables, while Mann–Whitney U tests were employed for variables that did not conform to normality assumptions. The FDR algorithm was applied for multiple comparisons to control the likelihood of generating false positives. The significance threshold was set at *p* = 0.05.

## Conflict of Interest

The authors declare no conflict of interest.

## Author Contributions

B.L., Z. Y., Z.C., Z. Y., Y.Y., and W.J. contributed equally to this work. Conception and design were performed by B.L., M.W., R.G., and Y.Z. Collection and assembly of data were done byZ.Y., Z.C., Z.Y., Y.Y., S.G., C.J., C.S., Z.W., Z.C., and Y.X. B.L. and M.W. drafted the article. Financial support was provided by R.G., Q.Z., Z.M., N.Q., W.J., and Y.Z. Critical revision of the article was done by L.C., W.J., V.M., P.Z., X.S., X.C., X.Z., L.Z., M.H., Y.W., H.Y., Y.L., and Z.Z.

## Supporting information

Supporting Information

Supporting Information

Supporting Information

## Data Availability

The data that support the findings of this study are available from the corresponding author upon reasonable request.
