# Supplementary material for: Integrated Microbiome and Metabolome Analysis Reveals Hypothalamic‐Comorbidities Related Signatures in Craniopharyngioma
Source: Adv Sci (Weinh). 2024 Sep 3;11(39):2400684. doi: 10.1002/advs.202400684 (PMC11497089; doi:10.1002/advs.202400684)
Supplement: Supplementary file 1 — Supporting Information [file ADVS-11-2400684-s002.docx]

**Supporting Information**

**Integrated microbiome and metabolome analysis reveals hypothalamic-comorbidities related signatures in craniopharyngioma**

*Ben Lin^†^, Zhen Ye^†^, Zhan Cao^†^, Zhao Ye^†^, Yifei Yu^†^, Weiliang Jiang ^†^, Sichen Guo, Vladimir Melnikov, Peng Zhou, Chenxing Ji, Chengzhang Shi, Zerui Wu, Zhengyuan Chen, Yihua Xu, Qilin Zhang, Zengyi Ma, Nidan Qiao, Long Chen, Xuefei Shou, Xiaoyun Cao, Xiang Zhou, Li Zhang, Min He, Yongfei Wang, Hongying Ye, Yiming Li, Zhaoyun Zhang, Meng Wang^*^, Renyuan Gao^*^ and Yichao Zhang^*^*

**^†^These authors contributed equally to this work.**

**^*^Corresponding authors**

**Clinical data**

Clinical data encompassed demographic features (age, sex, body weight, and Body Mass Index (BMI)), history of hypertension and diabetes mellitus, clinical manifestations (visual impairment, memory disturbance, hypopituitarism, and central diabetes insipidus), tumor features (MR classification, histopathology, and Ki67), abdominal ultrasound, and laboratory testing. BMI was calculated using the formula: BMI = weight (kg)/height^2^ (m^2^).

MR images were independently graded based on the degree of hypothalamic involvement, categorized as follows: grade 0, no hypothalamic involvement; grade 1, tumor abutting or displacing the hypothalamus; and grade 2, hypothalamic involvement to the extent that the hypothalamus could no longer be identifiable.^[1]^

Neurocognitive assessment in this study employed two general questionnaires: the Short Form 36 (SF-36) and Symptom Check List-90 (SCL90). The Chinese version of the SF-36, adapted from the International Quality of Life Assessment (IQOLA) SF-36 Standard UK Version 1.0, was employed.^[2]^ The questionnaire comprised a single health transition (HT) item and 35 items categorized into eight subscales: (1) physical function, (2) role physical (limitations due to physical health problems), (3) bodily pain (BP), (4) general health, (5) vitality, (6) social functioning, (7) role emotional (limitations due to emotional health problems), and (8) mental health. Higher z-transformed scores indicated better functional status, while lower scores indicated poorer Quality of Life.^[3]^

The SCL-90, a 90-item self-report symptom inventory, evaluated psychological distress and psychopathological symptoms. Inventory items were categorized into nine dimensions: somatization, obsessive-compulsive, interpersonal sensitivity, depression, anxiety (ANX), hostility (HOS), phobic ANX, paranoid ideation, and psychoticism.^[4]^ The Global Severity Index (GSI), calculated as the mean of all 90 items on the SCL-90, was further converted into a t-score (mean = 50, SD = 10) to measure composite psychological distress.^[5]^ A higher GSI T-score generally indicated an increased risk of psychological symptoms.^[3, 6]^

The diagnosis of NAFLD in CP patients was established according to literatures.^[7]^ Patients with positive hepatitis B or C serology or with evidence of inherited, autoimmune, cholestatic, drug-induced, or metabolic liver disease were excluded using standard clinical, laboratory, imaging, and histologic criteria. And patients with a weekly alcohol intake of 140 g or more were excluded. Diagnosis of NAFLD was determined by fatty infiltration on ultrasound analysis in association with abnormal liver enzymes. In addition, patients were excluded if they had a secondary cause of NAFLD.

**Norm of Chinese population**

The norms of SF-36 and SCL90 in Chinese population were based on the studies conducted by Wang et al.^[8]^ and Jin et al.^[9]^ respectively.

**Collection and processing of samples**

All participants in this study, including both the CP and HC groups, underwent equivalent fasting periods before sample collection. Fecal and serum samples, collected for correlation analysis, were obtained on the same day. Upon admission to the ward, patients were instructed to fast overnight, after which biological samples (feces and blood) were collected the following morning. Notably, no treatments were administered to patients during the sample collection process. Samples from the HC group were collected from the community and processed following the same protocol as for the CP group.

Fecal samples were collected using Sarstedt feces collection containers (SARSTEDT, Germany, cat. no.: 80.734.311) in accordance with the manufacturer's guidelines. These samples were then transferred to the laboratory on ice within 1 h and promptly stored at –80°C until further processing. It is worth mentioning that fecal samples were consistently collected by the same staff members.

For fasting plasma samples, all subjects provided samples that were immediately subjected to centrifugation at 7,500 × *g* for 20 min. The resulting supernatant was stored at –80°C for subsequent metabolomic analysis. To precipitate proteins, methanol/acetonitrile (1:1) was employed as the extraction solvent. In preparation for analysis, 200 μL of methanol/acetonitrile (1:1) was added to 50 μL of plasma, followed by a 10 s vortex. This mixture was then held at –20°C for a minimum of 2 h, followed by a 20 min centrifugation at 12,000 rpm. The supernatant was subsequently transferred to a clean tube for HPLC–MS analysis.^[10]^

**Tamoxifen administration**

To induce genetic recombination in Rax-CreER^T2^::Braf^V600E/+^::Pten^Flox/+^, adult mice received intraperitoneal injections of tamoxifen (T5648; Sigma–Aldrich) at a dose of 132 mg/kg body weight for three consecutive days. A stock of tamoxifen (66 mg/mL; Sigma; T5648) was prepared in a 5:1 ratio of corn oil to ethanol at 37°C with occasional vortexing, following established protocols.

**Mouse magnetic resonance imaging**

In vivo verification of tumor formation was conducted through MRI at the 20th week following tamoxifen administration. The MRI experiments were performed utilizing an 11.7 T Biospec small-animal MRI scanner (Bruker Corporation, Billerica, MA, USA) equipped with a 50 mm diameter transmit-receive volume coil. During the MRI scans, animals were gently anesthetized with 1.5–2% isoflurane in oxygen (1 L/min). T2-weighted imaging was employed in this study for improved visualization of brain lesions. The MRI experiments were supported by Zhangjiang Brain Imaging Center.

**Morris water maze**

Cognitive function assessment employed the Morris water maze test. Over four consecutive days, mice underwent five trials each day. In all trials, mice were introduced into a pool (120 cm in diameter and 50 cm in height) containing a 10 cm-diameter platform submerged 1 cm below the water's surface in quadrant III. The mice navigated the pool to locate the hidden platform, relying on visual cues around the pool for memory. Each mouse was placed in the water facing the pool wall and allowed 60 s to find the hidden platform. If a mouse failed to reach the platform within 60 s, the escape latency was recorded as 60 s. On the fifth day, a 60 s probe trial was conducted to assess the mice's memory. Parameters including the number of times mice crossed the platform area, distance traveled, and time spent in the target quadrant (quadrant III) were recorded with a digital video camera.

**Mouse gut microbiome**

Stool samples were promptly collected and frozen at –80°C within 2 h after sampling. The processing procedure closely resembled that described for the 16S rRNA gene sequencing of clinical patients. Specifically, alpha diversity (Shannon index) was analyzed at the OTU level using the Kruskal–Wallis test. PLS-DA analysis relied on the weighted UniFrac distance algorithm. Significant differences in relative abundance at the genus level were assessed using the Kruskal–Wallis rank-sum test. Linear discriminant analysis (LDA) effect size (LEfSe)^[11]^ was employed to identify significantly different species among different groups, with LDA providing an estimate of the effect of each species' abundance on these differences.

Regarding gut metagenomics, the processing procedure closely resembled that described for the metagenomic sequencing of clinical patients. After taxonomic and functional annotation, we examined the intra-group alterations of *Clostridium*, purine metabolism pathway and its related genes. The Wilcoxon test function from the stats package in R was employed for analyzing differences between two groups, while the Kruskal test function from the same package in R was used for analyzing differences among more than two groups.

**Mouse serum parameters and metabolomics**

After MRI scanning, the animals were humanely euthanized under anesthesia, and serum and liver samples were prepared. An automatic biochemical analyzer (SIEMENS, ADVIA XPT) was used to perform serum liver function tests, lipid profile analysis, and renal function tests. The workflow for metabolomics measurements and analyses remained consistent with the previously described procedure. Liver tissues were fixed in 4% paraformaldehyde and embedded in paraffin (Servicebio Technology), followed by staining with a Masson trichrome solution.

**mRNA isolation and quantitative real‐time polymerase chain reaction**

Total mRNA was extracted from liver and brain cortex with Trizol reagent (Invitrogen, USA). The product was quantified by NanoDrop One/Onec (Thermo Scientific). Reverse transcription and gDNA removal were achieved by AdvanceFast One-step RT-gDNA Digestion SuperMix for qPCR (YEASEN) on ice. The reaction program was run by ProFlex™ (Thermo Scientific). The amplification system of qPCR was built by qPCR SYBR Green Master Mix (YEASEN). The amplification program was run by Quantstudio Dx (ABI). The primers were synthesized by Sangon Biotech (Shanghai, China). All data were analyzed using the 2−ΔΔCT method and normalized with GADPH or Actin. Primer sequences are listed in **Supplementary Table 9**.

**Statistical analysis of sample size in animal experiments**

To examine the fidelity of the results obtained from our animal studies, we performed statistical analysis of phenotypic results (post hoc analysis and/or one-way ANOVA analysis). In the pilot animal study (**Supplementary Table 10**), post hoc analysis of water maze experiment demonstrated limited power (1 – β error probability = 0.161) despite statistically significant variation, and post hoc analysis of hepatic Masson staining indicated satisfying power (1 – β error probability = 0.878). With respect to intervention experiments focused on hypoxanthine (**Supplementary Table 11**), the post hoc analysis (1 – β error probability = 0.999) and ANOVA analysis of different treatments (SS = 23776, DF = 3, MS = 7925, F = 15.92, P < 0.0001) in water maze experiment revealed satisfying power and indicated that the inter-group variance is significantly larger than intra-group variance. Regarding hepatic pathology, the post hoc analysis (1 – β error probability = 0.999 for both Masson staining and Oil-red O staining) and the ANOVA analysis of different treatments (SS = 69.57, DF = 3, MS = 23.19, F = 33.33, P < 0.0001 for Masson staining; SS = 266.8, DF = 3, MS = 88.92, F = 56.35, P < 0.0001 for Oil-red O staining) demonstrated satisfying power and indicated that the inter-group variance is significantly larger than intra-group variance.

**References**

[1] S. Puget, M. Garnett, A. Wray, J. Grill, J. L. Habrand, N. Bodaert, M. Zerah, M. Bezerra, D. Renier, A. Pierre-Kahn, C. Sainte-Rose, *J Neurosurg* **2007**, *106* (1 Suppl), 3, <https://doi.org/10.3171/ped.2007.106.1.3>.

[2] L. Li, H. Wang, Y. Shen, *Zhonghua Yu Fang Yi Xue Za Zhi* **2002**, *36* (2), 109.

[3] Y. Lin, Y. Yu, J. Zeng, X. Zhao, C. Wan, *Health Qual Life Outcomes* **2020**, *18* (1), 360, <https://doi.org/10.1186/s12955-020-01605-8>.

[4] L. R. Derogatis, R. S. Lipman, L. Covi, *Psychopharmacol Bull* **1973**, *9* (1), 13.

[5] F. Monteiro, M. C. Canavarro, M. Pereira, *Psychol Health Med* **2017**, *22* (9), 1105, <https://doi.org/10.1080/13548506.2017.1281972>.

[6] F. Tian, H. Li, S. Tian, J. Yang, J. Shao, C. Tian, *Psychiatry Res* **2020**, *288*, 112992, <https://doi.org/10.1016/j.psychres.2020.112992>.

[7] a) L. A. Adams, A. Feldstein, K. D. Lindor, P. Angulo, *Hepatology* **2004**, *39* (4), 909, <https://doi.org/10.1002/hep.20140>; b) A. Hoffmann, K. Bootsveld, U. Gebhardt, A. M. Daubenbuchel, A. S. Sterkenburg, H. L. Muller, *Eur J Endocrinol* **2015**, *173* (3), 389, <https://doi.org/10.1530/EJE-15-0422>.

[8] W. Rui, W. Cheng, X. Q. Ma, Y. F. Zhao, X. Y. Yan, H. Jia, *Scand J Public Health* **2011**, *39* (4), 410, <https://doi.org/10.1177/1403494810395817>.

[9] H. Jin, W. Y. Wu, M. Y. Zhang, *Chinese Journal of Nervous and Mental Diseases* **1986**, *12* (5), 260.

[10] Y. Fan, Y. Li, Y. Chen, Y. J. Zhao, L. W. Liu, J. Li, S. L. Wang, R. N. Alolga, Y. Yin, X. M. Wang, D. S. Zhao, J. H. Shen, F. Q. Meng, X. Zhou, H. Xu, G. P. He, M. D. Lai, P. Li, W. Zhu, L. W. Qi, *J Am Coll Cardiol* **2016**, *68* (12), 1281, <https://doi.org/10.1016/j.jacc.2016.06.044>.

[11] N. Segata, J. Izard, L. Waldron, D. Gevers, L. Miropolsky, W. S. Garrett, C. Huttenhower, *Genome Biol* **2011**, *12* (6), R60, <https://doi.org/10.1186/gb-2011-12-6-r60>.

**Figure S1. 16s ribosomal RNA gene sequencing showed an altered microbial composition in CP patients.**

**(A)** A venn diagram showed that 713 of the 1070 OTUs were consistent between CP group (n = 57) and HC group (n = 67), while 253 and 104 OTUs were exclusively found in CP group and HC group, respectively. **(B)** Alpha diversity was estimated by the chao 1 index (p = 0.64), goods coverage index (p = 0.39), PD whole tree index (p = 0.82), observed species index (p = 0.69), shannon index (p = 0.077) and simpson index (p = 0.053) between patients with CP and healthy controls. **(C, D)** The taxonomic distributions in each group at phylum (C) and genus (D) clades.

**Figure S2. 16S rRNA gene sequencing analysis of fecal samples from CP patients and Healthy controls.**

**(A, B)** Beta diversity of craniopharyngioma patients (CP, n = 57) vs healthy controls (HC, n = 67) was calculated using weighted Unifrac and analyzed by ANOSIM (A) and ADONIS (B), respectively. **(C)** RDA analysis was utilized to assess the potential confounding effect of the basic information and the permutation test indicated non-significant effect (p = 0.091) effect from the combination of these baseline indices. **(D)** The significantly differential genus in CP patients (blue) and healthy individuals (orange) detected by Wilcoxon Rank-Sum Test. Only the 20 most abundant species are shown.

**Figure S3.** **Shotgun metagenomics sequencing showed an altered microbial composition at genus level.**

**(A)** Alpha diversity at genus level of craniopharyngioma patients (CP, n = 56) vs healthy controls (HC, n = 52) was calculated using Shannon-Wiener index (p < 0.001). ^***^ denotes p<0.001. **(B, C)** Beta diversity at genus level was calculated using weighted UniFrac and analyzed by ANOSIM **(B)** and ADONIS **(C)** , respectively. **(D)** The Venn diagram showed that 1564 genus were shared between the CP group and HC group, while 96 and 332 genera were exclusively found in CP group and HC group respectively. **(E)** The taxonomic distributions in each group at phylum (upper) and genus (lower) clades. **(F)** The differential genus between CP patients (blue) and healthy individuals (purple) detected by Wilcox Rank-Sum Test. Only the 10 most abundant species in each group are shown.

**Figure S4. Functional differences and annotated pathways in CP patients.**

**(A)** The significantly functional differences in CP patients (purple, n = 56) and healthy individuals (blue, n = 52) detected by Wilcoxon Rank-Sum Test. Only the 20 most abundant functional differences in each group are shown. **(B)** The barplot displays the number of KOs occupied by each pathway in both groups, with carbohydrate metabolism, global and overview maps, amino acid metabolism, energy metabolism, metabolism of cofactors and vitamins, nucleotide metabolism and lipid metabolism enriched in CP group.

**Figure S5. Serum metabolomics indicated significant alteration in CP patients.**

**(A, B)** PCA score plots of plasma samples from patients (n = 52) with CP and healthy controls (HC, n = 50) in positive-mode (POS, A) and negative-mode (NEG, B) showed a slight separation in two groups. **(C, D)** OPLS-DA score plots in positive-mode (POS, C) and negative-mode (NEG, d) exhibited a clear separation in the two groups. **(E, F)** S-plots in OPLS-DA positive-mode (POS, E) and OPLS-DA negative-mode (NEG, F). **(G)** The pie plot presented the enriched chemical class after aligning the differential metabolites against the Pubchem database. **(H)** The boxplots presented the relative abundance of hypoxanthine, 2-methoxyestrone 3-glucuronide, eicosapentaenoic acid and 3-hydroxylidocaine, which were annotated to purine metabolism (CP upregulated↑), steroid hormone biosynthesis (CP downregulated↓), biosynthesis of unsaturated fatty acids (CP upregulated↑) and drug metabolism - cytochrome P450 (CP upregulated↑) respectively. **(I, J)** The Sankey plots demonstrate the potential biological reactions (R01863 and R02748) by which the gut microbiome exerts their influences on serum hypoxanthine level, with each node presenting a taxon probably involved in the biological reaction.

**Figure S6. Alterations of phenotypes and gut microbiome in transgenic rodent model with CP.**

**(A, B, C)** There were no significant differences in the parameters of the memory phase between tumor group (n = 3) and control group (n = 6). Liver functions **(D)** and renal functions **(E)** showed no significant alterations between tumor group and control group. **(F)** Ternary analysis indicated that the pre-tumor formation rodent model and wild-type control were characterized by the genera *Lactobacillus* and *Faecalibaculum*, respectively. **(G)** Kruskal–Wallis test revealed that the post-tumor formation rodent model was characterized with an increased abundance of *Clostridium innocuum*.

**Figure S7. Perturbed purine metabolism, increased *Clostridium* and dysregulated anti-oxidative system are correlated with the development and progression of CP-associated hypothalamic comorbidities.**

**(A)** The histopathological analyses (Masson staining and Oil red O staining) indicate intensified fibrosis and elevated lipid accumulation in the CP mice treated with WD. **(B)** Quantitative analyses of histopathological results reveal a notable increase in collagen volume fraction and oil red area fraction in CP mice treated with WD. **(C)** The line chart of the water maze demonstrates that CP mice in WD group exhibited a significantly prolonged escape latency on the last day of the learning phase compared with Control + WD group. **(D)** The representative trajectory diagram of each group in the water maze experiment. **(E)** Compared with Control+ WD group, the hepatic mRNA expression of critical enzymes in purine metabolism (HPRT and XOR) as well as crucial players in anti-oxidative system (SOD-1 and CAT) are notably increased in Tumor + WD group. **(F)** Similarly, the cortical mRNA expression of critical enzymes in purine metabolism (HPRT, XOR and PNP) and crucial players in anti-oxidative system (Nrf2, SOD-1 and CAT) are notably increased in Tumor + WD group. **(G)** The bar chart displays an increased Shannon index in the Tumor + WD group compared to the Control+ WD group (p = 0.352). **(H)** Anosim analysis detects altered beta diversity between Tumor + WD group compared to the Control+ WD group (R^2^ = 0.132, p = 0.115). **(I)** Wilcoxon rank-sum test demonstrated that *Clostridium sensu stricto 1* is notably enriched in Tumor + WD group. WD: western diet; CD: chow diet; HPRT: Hypoxanthine-guanine phosphoribosyl transferase; XOR: Xanthine oxidoreductase; PNP: Purine nucleoside phosphorylase; Nrf2: Nuclear factor erythroid 2-related factor 2; SOD-1: Superoxide dismutase 1; CAT: Catalase; *p < 0.05, **p < 0.01, ***p < 0.001, ****p < 0.0001. N = 5 in each group.

**Supplementary Table 1. Baseline demographic information of CP and HC participants of 16s rRNA sequencing**

|  |  | CP(n = 57) | HC(n = 67) | p value |
| --- | --- | --- | --- | --- |
| Sex | female | 21(36.8%) | 39(58.2%) | 0.018 |
|  | male | 36(63.2%) | 28(41.8%) |  |
| Age (years) |  | 43.81±16.50 | 48.22±2.96 | 0.051 |
| BMI (kg/cm2) |  | 24.26±4.98 | 23.44±2.94 | 0.263 |
| HBP |  | 10(17.5%) | 8(11.9%) | 0.377 |
| DM |  | 2(3.5%) | 3(4.5%) | 1.000 |

BMI: body mass index; HBP: hypertension; DM: diabetes mellitus

**Supplementary Table 2. Baseline demographic information of CP and HC participants of metagenomic sequencing**

|  |  | CP(n = 56) | HC(n = 52) | p value |
| --- | --- | --- | --- | --- |
| Sex | female | 20(35.7%) | 14(26.9%) | 0.326 |
|  | male | 36(64.3%) | 38(73.1%) |  |
| Age (years) |  | 43.66±16.61 | 40.54±13.69 | 0.291 |
| BMI (kg/cm2) |  | 24.69±4.21 | 23.08±3.19 | 0.028 |
| HBP |  | 10(17.9%) | 7(13.5%) | 0.531 |
| DM |  | 2(3.6%) | 2(3.8%) | 1.000 |

BMI: body mass index; HBP: hypertension; DM: diabetes mellitus

**Supplementary Table 3. Baseline characteristics of these 57 patients with craniopharyngioma**

|  | Total (n = 57) |  |  |  |
| --- | --- | --- | --- | --- |
| **Demographic features** |  |  |  |  |
| Female (n, %) | 21, 36.84% |  |  |  |
| Age (years) | 43.8±2.185 |  |  |  |
| BMI (kg/cm^2^) | 24.6±0.555 |  |  |  |
| SBP (mmHg) | 119.2±2.003 |  |  |  |
| DBP (mmHg) | 76.6±1.397 |  |  |  |
| **Tumor Features** |  |  |  |  |
| Hypothalamic involvement（n, %） | 48, 84.21% | Puget 0  (n= 9, 15.79%) | Puget 1  (n= 15, 26.32%) | Puget 2  (n= 33, 57.89%) |
| Histopathology（n, %） |  | ACP  (n= 43, 75.44%) | PCP  (n=12, 21.05%) | Cystic Lesions  (n=2, 3.51%) |
| Ki67 (%) | 3.5±0.265 |  |  |  |
| **Fatty liver (n = 33)** | 16, 48.48% |  |  |  |
| **Medication history** |  |  |  |  |
| Antihypertensive drug | 10, 17.5% |  |  |  |
| Hypoglycemic drug | 2, 3.5% |  |  |  |
| **Laboratory testing** |  |  |  |  |
| WBC（x10^9/L） | 6.2±0.201 |  |  |  |
| NEU (%) | 3.5±0.153 |  |  |  |
| LYM (%) | 2.2±0.090 |  |  |  |
| MON (%) | 0.4±0.017 |  |  |  |
| ALP (U/L) | 69.9±3.392 |  |  |  |
| ALB (g/L) | 44.2±0.473 |  |  |  |
| TBIL (umol/L) | 9.0±0.671 |  |  |  |
| UA (mmol/L) | 0.3±0.013 |  |  |  |
| DBIL (umol/L) | 3.7±0.407 |  |  |  |
| BUN (mmol/L) | 4.6±0.192 |  |  |  |
| CRE (umol/L) | 71.1±2.081 |  |  |  |
| GGT (U/L) | 41.4±6.557 |  |  |  |
| TPRO (g/L) | 68.5±0.691 |  |  |  |
| ALT (U/L) | 35.7±5.681 |  |  |  |
| AST (U/L) | 27.2±2.652 |  |  |  |
| HbA1c (%) | 5.8±0.081 |  |  |  |
| FPG (mmol/L) | 4.9±0.085 |  |  |  |
| FINS (mU/L) | 14.2±3.032 |  |  |  |
| FC-PR (ug/L) | 2.6±0.212 |  |  |  |
| 2hPG (mmol/L) | 8.3±0.541 |  |  |  |
| 2hINS (mU/L) | 101.6±15.32 |  |  |  |
| 2hC-PR (ug/L) | 10.2±0.756 |  |  |  |
| TG (mmol/L) | 1.9±0.216 |  |  |  |
| CHO (mmol/L) | 5.1±0.196 |  |  |  |
| sdLDL (mmol/L) | 3.3±0.183 |  |  |  |
| HCY (umol/L) | 12.2±0.934 |  |  |  |
| GH (ng/mL) | 0.4±0.060 |  |  |  |
| IGF-1 index | 0.5±0.027 |  |  |  |
| ACTH (pg/mL) | 53.7±4.652 |  |  |  |
| TSH (mIU/L) | 1.8±0.165 |  |  |  |
| TT4 (nmol/L) | 89.4±2.439 |  |  |  |
| TT3 (nnol/L) | 1.5±0.046 |  |  |  |
| FT4 (pmol/L) | 13.1±0.410 |  |  |  |
| FT3 (pmol/L) | 4.2±0.119 |  |  |  |
| FSH (IU/L) | 6.2±1.015 |  |  |  |
| LH (IU/L) | 3.8±0.532 |  |  |  |
| PRL (ng/mL) | 38.3±4.572 |  |  |  |
| DHEA (umol/L) | 5.5±0.523 |  |  |  |
| COR (ug/dl) | 11.2±0.875 |  |  |  |
| E2 (pmol/L) | 80.3±5.532 |  |  |  |
| T (nmol/L) | 5.3±0.899 |  |  |  |
| PRO (nmol/L) | 0.6±0.053 |  |  |  |

SBP: systolic blood pressure; DBP: diastolic blood pressure; WBC: white blood cell; NEU: neutrophil; LYM: lymphocyte; MON: monocyte; ALP: alkaline phosphatase; ALB: albumin; TBIL: total bile acid; UA: uric acid; DBIL: direct bile acid; BUN: blood urea nitrogen; CRE: creatinine; GGT: Gamma-glutamyl transpeptidase; TPRO: total protein; ALT: alanine transaminase; AST: aspartate transaminase; HbA1c: Hemoglobin A1c; FPG: fasting plasma glucose; 2hPG: 2-hour postprandial plasma glucose; 2hINS: 2-hour postprandial insulin; 2h C peptide: 2-hour postprandial C-peptide; TG: triglyceride; CHO: cholesterol; sdLDL: small dense low density lipoprotein; HCY: homocysteine; GH: growth hormone; IGF-1: insulin like growth factor 1; ACTH: adrenocorticotropic hormone; TSH: thyroid stimulating hormone; T4: total thyroxine; T3: total triiodothyronine; FT4: free thyroxine; FT3: free triiodothyronine; LH: luteinizing hormone; PRL: prolactin; DHEA: dehydroepiandrosterone; COR: cortisol; FSH: follicle stimulating hormone; E2: estradiol; T: testosterone; P: progesterone; PRO: progesterone.

**Supplementary Table 4. Parameters of neurocognitive questionnaires between patients with different Puget grades**

|  |  | Chinese Norm | CP patients | Puget 1(n = 11) | Puget 2(n = 16) | p value |
| --- | --- | --- | --- | --- | --- | --- |
| **SF-36** | Physical functioning | 94.02±12.44 | 81.30±20.97 | 87.73±14.21 | 76.88±24.01 | ^*^<0.001  ^#^0.192 |
|  | Role physical | 88.79±28.49 | 48.15±45.96 | 52.27±48.03 | 45.31±45.84 | ^*^<0.001  ^#^0.707 |
|  | Bodily pain | 88.18±19.02 | 69.96±19.94 | 69.18±20.84 | 70.50±19.97 | ^*^<0.001  ^#^0.870 |
|  | General health | 69.74±20.95 | 60.41±20.45 | 62.64±24.01 | 58.88±18.28 | ^*^0.193  ^#^0.648 |
|  | Vitality | 68.92±18.78 | 57.41±16.78 | 57.73±17.37 | 57.19±16.93 | ^*^0.018  ^#^0.936 |
|  | Social functioning | 88.03±16.00 | 94.91±24.58 | 94.32±24.60 | 95.31±25.36 | ^*^0.213  ^#^0.920 |
|  | Role emotional | 89.57±27.95 | 39.51±45.33 | 48.49±47.99 | 43.89±33.33 | ^*^<0.001  ^#^0.404 |
|  | Mental health | 77.61±15.85 | 65.33±16.79 | 64.00±17.25 | 66.25±16.97 | ^*^<0.001  ^#^0.739 |
| **SCL90** | Somatization | 1.37±0.48 | 1.63±0.60 | 1.80±0.788 | 1.51±0.415 | ^*^0.060  ^#^0.231 |
|  | Compulsion | 1.62±0.58 | 1.65±0.53 | 1.67±0.524 | 1.64±0.555 | ^*^0.952  ^#^0.870 |
|  | Interpersonal relationship | 1.65±0.51 | 1.43±0.45 | 1.53±0.509 | 1.36±0.403 | ^*^0.213  ^#^0.359 |
|  | Depression | 1.50±0.59 | 1.61±0.49 | 1.62±0.510 | 1.61±0.489 | ^*^0.914  ^#^0.933 |
|  | Anxiety | 1.39±0.43 | 1.46±0.58 | 1.60±0.800 | 1.36±0.359 | ^*^0.918  ^#^0.373 |
|  | Hostility | 1.48±0.56 | 1.45±0.36 | 1.52±0.320 | 1.41±0.394 | ^*^0.952  ^#^0.455 |
|  | Phobia | 1.23±0.41 | 1.28±0.47 | 1.43±0.664 | 1.17±0.251 | ^*^0.918  ^#^0.241 |
|  | Paranoia | 1.43±0.57 | 1.28±0.38 | 1.33±0.500 | 1.24±0.272 | ^*^0.737  ^#^0.534 |
|  | Psychoticism | 1.29±0.42 | 1.36±0.50 | 1.36±0.650 | 1.35±0.400 | ^*^0.918  ^#^0.947 |

SF-36: Short Form 36; SCL90: Symptom Check List-90; GSI: global severity; ^*^: CP patients compared with Chinese norm; ^#^: Puget grade 2 compared with Puget grade 1.

**Supplementary Table 5. Significantly altered genera between CP group and HC group detected by multivariate association with linear models (MaAsLin)**

| Genus | Coefficient | q value |
| --- | --- | --- |
| *Eubacterium* | -0.180339034 | 0.000000000187 |
| *Blautia* | -0.075595563 | 0.00000000139 |
| *Clostridium* | -0.16609418 | 0.000000531 |
| *Ruminococcus* | -0.082410702 | 0.00000552 |
| *Roseburia* | -0.073984728 | 0.0000450 |
| *Eubacterium_rectale* | -0.040647121 | 0.000708843 |
| *Alistipes* | 0.125944702 | 0.004856942 |
| *Prevotella* | 0.151020139 | 0.072246937 |
| *Sutterella* | 0.040034525 | 0.08829267 |

The positive coefficient value means the genus was enriched in HC group, and the negative coefficient value means the genus was enriched in CP group. Statistical analysis was performed using MaAsLin with adjustments for multiple comparisons (q value). CP: craniopharyngioma; HC: healthy control.

**Supplementary Table 6. Significantly altered species between CP group and HC group analyzed by multivariate association with linear models (MaAsLin)**

| Genus | Coefficient | q value |
| --- | --- | --- |
| *Eubacterium_eligens_CAG_72* | -0.121908181 | 0.000123931 |
| *Clostridium_sp_CAG_7* | -0.115852937 | 0.00051614 |
| *Eubacterium_rectale* | -0.072798773 | 0.000673913 |
| *Alistipes_putredinis* | 0.184292083 | 0.000673913 |
| *Eubacterium_sp_CAG_76* | -0.10735659 | 0.000806282 |
| *Alistipes_putredinis_CAG_67* | 0.104068734 | 0.001315432 |
| *Roseburia_inulinivorans* | -0.063663895 | 0.001513134 |
| *uncultured_Faecalibacterium_sp* | -0.08621564 | 0.003391646 |
| *Clostridium_sp_CAG_43* | -0.046808295 | 0.007404998 |
| *Alistipes_sp_CAG_53* | 0.075764651 | 0.009628639 |
| *Faecalibacterium_prausnitzii* | -0.102637353 | 0.022165062 |
| *Alistipes_shahii* | 0.054073142 | 0.0458436 |
| *Prevotella_stercorea* | 0.049965972 | 0.135161833 |
| *Clostridium_sp_CAG_127* | -0.047800584 | 0.202882783 |

The positive coefficient value means the genus was enriched in HC group, and the negative coefficient value means the species was enriched in CP group. Statistical analysis was performed using MaAsLin with adjustments for multiple comparisons (q value). CP: craniopharyngioma; HC: healthy control.

**Supplementary Table 7. Paired comparison in clinical indices of enrolled CP patients**

|  |  | Pre-operation | Follow-up | p value |
| --- | --- | --- | --- | --- |
| Sex | Female (18, 37.5%) |  |  |  |
|  | Male (30, 62.5%) |  |  |  |
| Age (years) | 44.79±15.96 |  |  |  |
| Follow-up months | 8.21±3.61 |  |  |  |
| BMI (kg/cm2) |  | 25.14±3.96 | 26.67±3.80 | <0.001 |
| FPG (mmol/L) |  | 4.97±0.66 | 4.82±0.70 | 0.232 |
| TG (mmol/L) |  | 1.95±1.57 | 2.24±1.37 | 0.254 |
| CHO (mmol/L) |  | 5.21±1.37 | 4.86±1.25 | 0.113 |
| Fatty liver (n = 33) |  | 16 (48.5%) | 26(81.3%) | 0.006 |

BMI: body mass index; FPG: fasting plasma glucose; TG: triglyceride; CHO: cholesterol

**Supplementary Table 10. Post hoc analysis to evaluate power of pilot animal study**

| Pilot study | Sample size group 1 | Sample size group 2 | Effect size | α error probability | Power (1 – β error probability) |
| --- | --- | --- | --- | --- | --- |
| Water maze | 6 | 3 | 0.785 | 0.05 | 0.161 |
| Hepatic Masson staining | 6 | 3 | 2.583 | 0.05 | 0.878 |

**Supplementary Table 11. Post hoc analysis to evaluate power of intervention animal study**

| Intervention study | Total sample size | Number of groups | Effect size | α error probability | Power (1 – β error probability) |
| --- | --- | --- | --- | --- | --- |
| Water maze | 17 | 4 | 1.813 | 0.05 | 0.999 |
| Hepatic Masson staining | 17 | 4 | 1.688 | 0.05 | 0.999 |
| Hepatic Oil-red O staining | 17 | 4 | 2.007 | 0.05 | 0.999 |
