# Supplementary material for: Integrated Microbiome and Metabolome Analysis Reveals Hypothalamic‐Comorbidities Related Signatures in Craniopharyngioma
Source: Adv Sci (Weinh). 2024 Sep 3;11(39):2400684. doi: 10.1002/advs.202400684 (PMC11497089; doi:10.1002/advs.202400684)

ADVANCED SCIENCE
Open Access

Supporting Information

for *Adv. Sci.*, DOI 10.1002/advs.202400684

Integrated Microbiome and Metabolome Analysis Reveals Hypothalamic-Comorbidities Related Signatures in Craniopharyngioma

Ben Lin, Zhen Ye, Zhan Cao, Zhao Ye, Yifei Yu, Weiliang Jiang, Sichen Guo, Vladimir Melnikov, Peng Zhou, Chenxing Ji, Chengzhang Shi, Zerui Wu, Zhengyuan Chen, Yihua Xu, Qilin Zhang, Zengyi Ma, Nidan Qiao, Long Chen, Xuefei Shou, Xiaoyun Cao, Xiang Zhou, Li Zhang, Min He, Yongfei Wang, Hongying Ye, Yiming Li, Zhaoyun Zhang, Meng Wang, Renyuan Gao* and Yichao Zhang**

Supplementary Figure 1

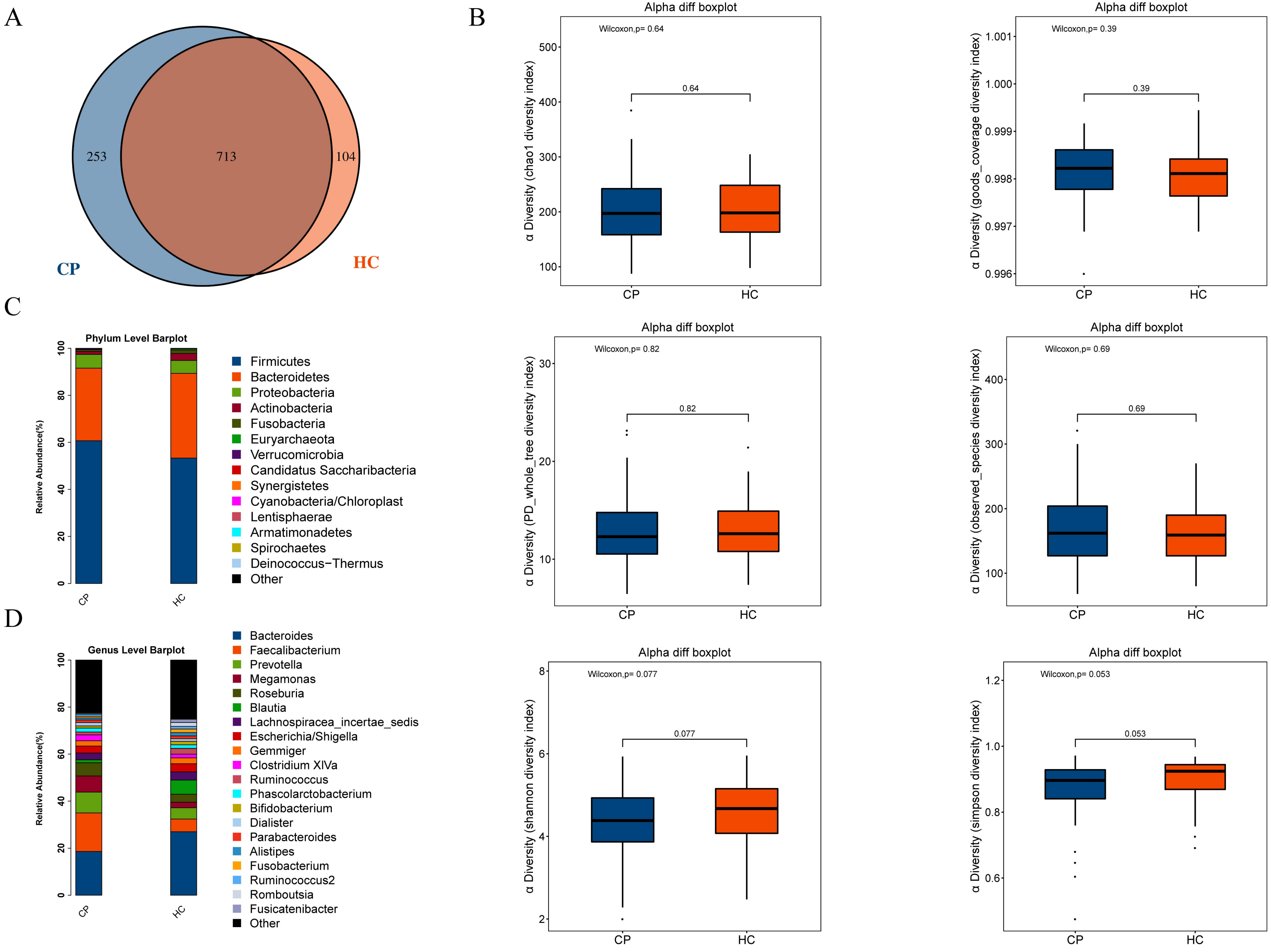

Supplementary Figure 2

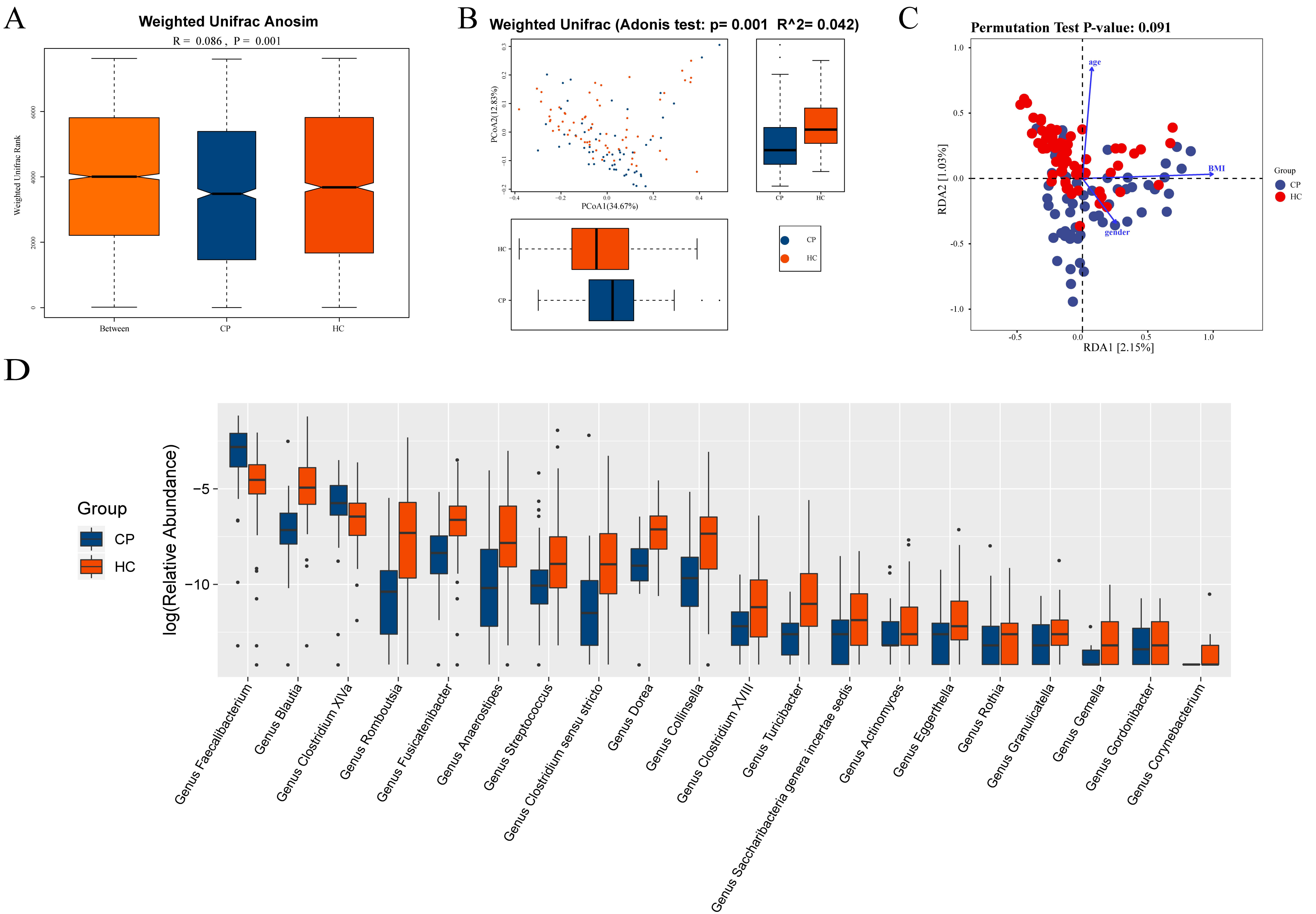

Supplementary Figure 3

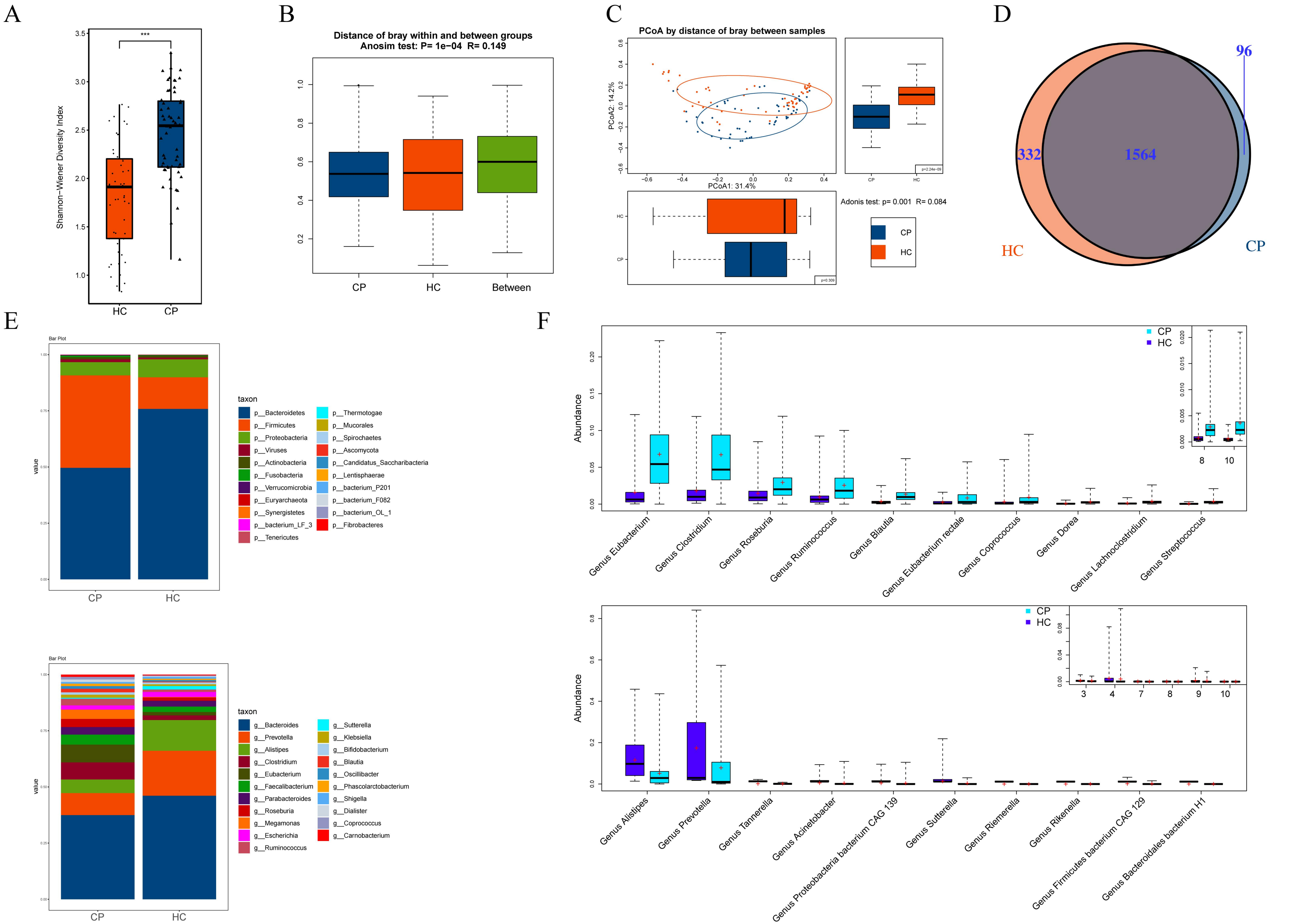

Supplementary Figure 4

A

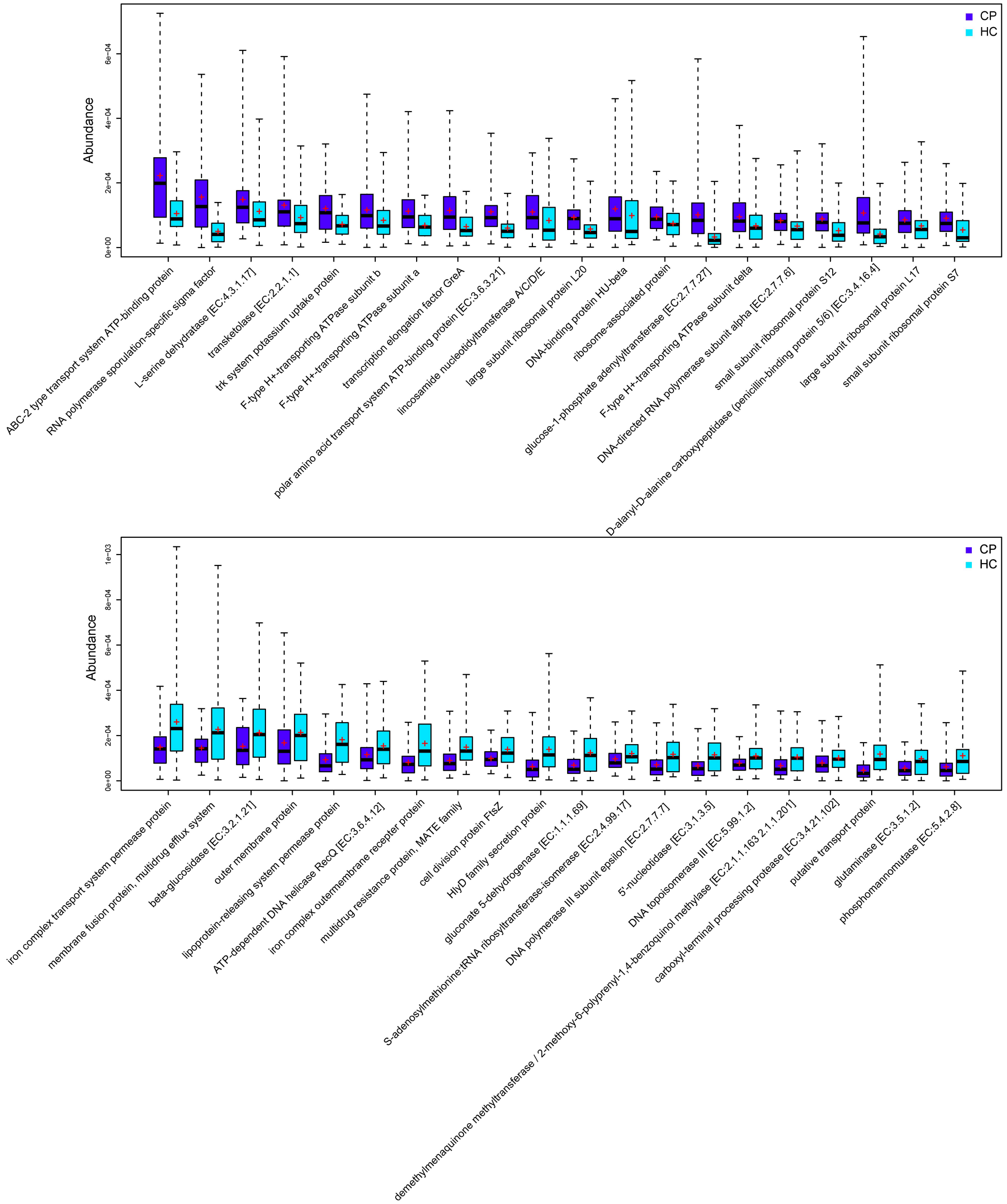

B

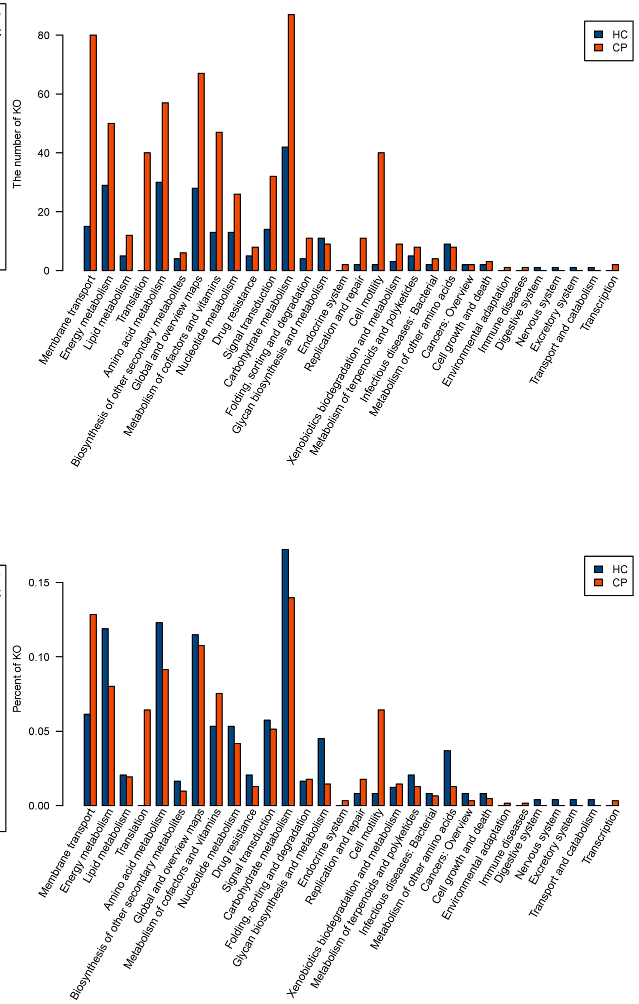

Supplementary Figure 5

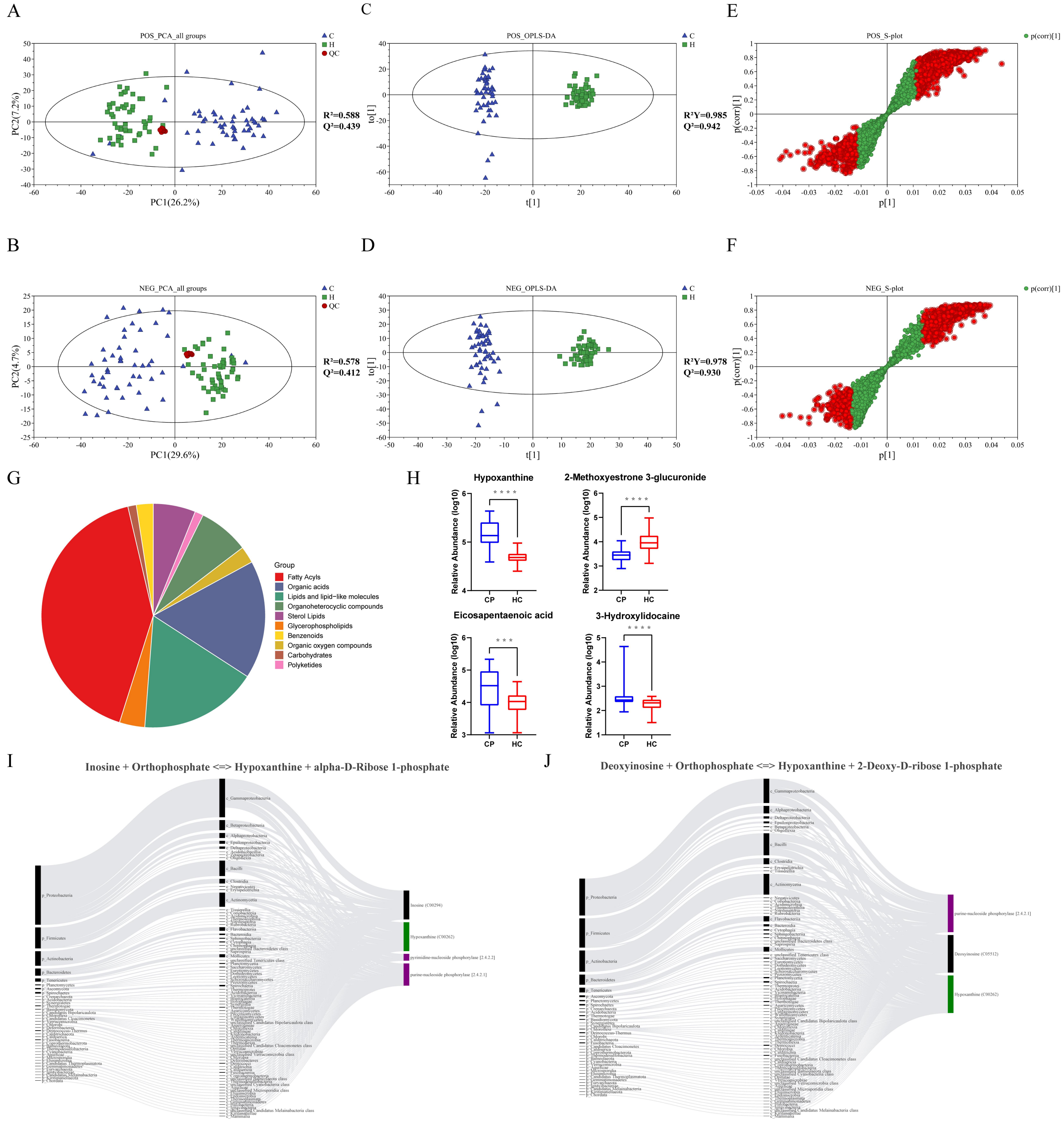

Supplementary Figure 6

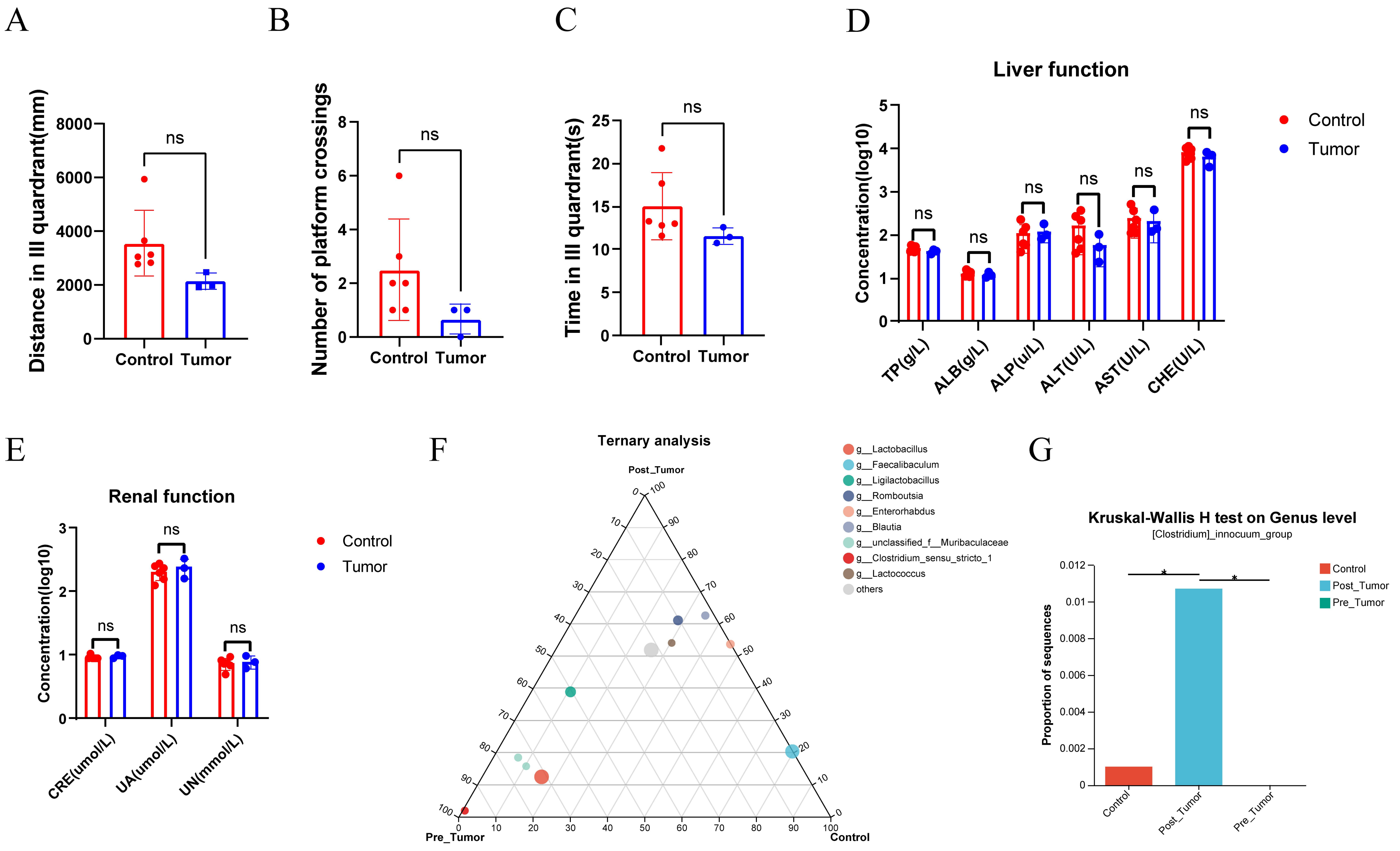

Supplementary Figure 7

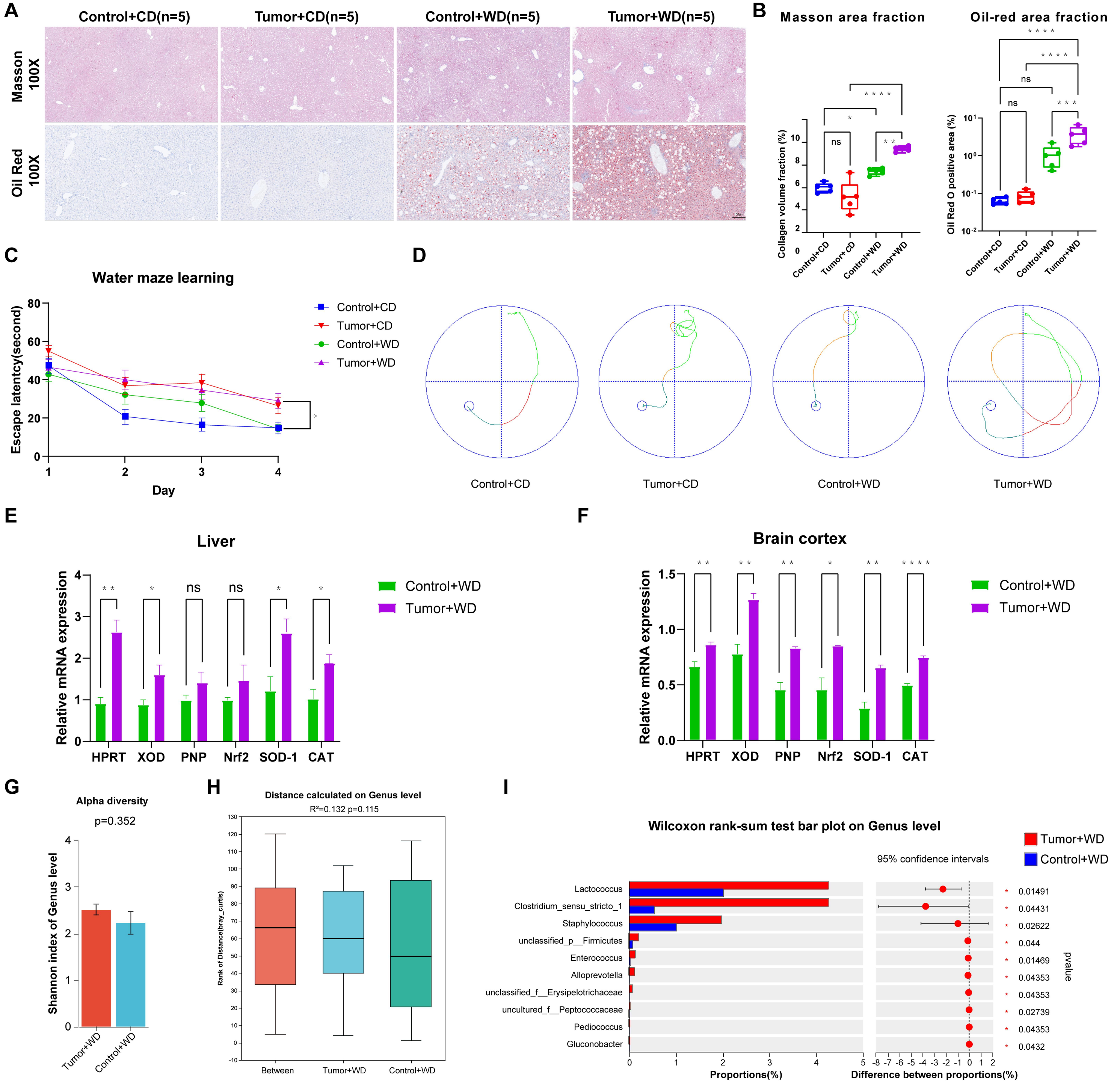

Supplement: Supplementary file 3 — Supporting Information [file ADVS-11-2400684-s001.pdf]
